# Update on infections caused by *Stenotrophomonas maltophilia* with particular attention to resistance mechanisms and therapeutic options

**DOI:** 10.3389/fmicb.2015.00893

**Published:** 2015-09-02

**Authors:** Ya-Ting Chang, Chun-Yu Lin, Yen-Hsu Chen, Po-Ren Hsueh

**Affiliations:** ^1^Division of Infectious Diseases, Department of Internal Medicine, Kaohsiung Municipal HsiaoKang HospitalKaohsiung, Taiwan; ^2^Division of Infectious Diseases, Department of Internal Medicine, Kaohsiung Medical University Hospital, Kaohsiung Medical UniversityKaohsiung, Taiwan; ^3^School of Medicine, Graduate Institute of Medicine, Sepsis Research Center, College of Medicine, Kaohsiung Medical UniversityKaohsiung, Taiwan; ^4^Department of Biological Science and Technology, College of Biological Science and Technology, National Chiao Tung UniversityHsinChu, Taiwan; ^5^Departments of Laboratory Medicine and Internal Medicine, National Taiwan University Hospital, National Taiwan University College of MedicineTaipei, Taiwan

**Keywords:** *Stenotrophomonas maltophilia*, prevalence, susceptibility, surveillance, treatment

## Abstract

*Stenotrophomonas maltophilia* is a Gram-negative, biofilm-forming bacterium. Although generally regarded as an organism of low virulence, *S. maltophilia* is an emerging multi-drug resistant opportunistic pathogen in hospital and community settings, especially among immunocompromised hosts. Risk factors associated with *S. maltophilia* infection include underlying malignancy, cystic fibrosis, corticosteroid or immunosuppressant therapy, the presence of an indwelling central venous catheter and exposure to broad spectrum antibiotics. In this review, we provide a synthesis of information on current global trends in *S. maltophilia* pathogenicity as well as updated information on the molecular mechanisms contributing to its resistance to an array of antimicrobial agents. The prevalence of *S. maltophilia* infection in the general population increased from 0.8–1.4% during 1997–2003 to 1.3–1.68% during 2007–2012. The most important molecular mechanisms contributing to its resistance to antibiotics include β-lactamase production, the expression of *Qnr* genes, and the presence of class 1 integrons and efflux pumps. Trimethoprim/sulfamethoxazole (TMP/SMX) is the antimicrobial drug of choice. Although a few studies have reported increased resistance to TMP/SMX, the majority of studies worldwide show that *S. maltophilia* continues to be highly susceptible. Drugs with historically good susceptibility results include ceftazidime, ticarcillin-clavulanate, and fluoroquinolones; however, a number of studies show an alarming trend in resistance to those agents. Tetracyclines such as tigecycline, minocycline, and doxycycline are also effective agents and consistently display good activity against *S. maltophilia* in various geographic regions and across different time periods. Combination therapies, novel agents, and aerosolized forms of antimicrobial drugs are currently being tested for their ability to treat infections caused by this multi-drug resistant organism.

*Stenotrophomonas maltophilia* is a Gram-negative, aerobic, glucose non-fermenting, motile bacillus. *S. maltophilia* was first isolated from pleural effusion in 1943 and initially named *Bacterium bookeri*. The organism was reclassified as a member of the genus *Pseudomonas* in 1961, *Xanthomonas* in 1983, and then *Stenotrophomonas* in 1993 (Al-Anazi and Al-Jasser, 2014). It survives on almost any humid surface and has been isolated from a wide variety of aquatic sources, such as suction tubing, nebulizers, endoscopes, hemodialysis dialysate samples, plant rhizosphere, faucets, sink drains, and shower heads (Brooke, 2012). S. *maltophilia* is characterized by its ability to form biofilms on various abiotic and biotic surfaces, including lung cells (de Oliveira-Garcia et al., 2003; Pompilio et al., 2010), and by its resistance to a broad array of antimicrobial agents. The World Health Organization recently classified S. *maltophilia* as one of the leading multidrug resistant organisms (MDROs) in hospital settings (Brooke, 2014).

*S. maltophilia* is generally regarded as an organism of low virulence and therefore an opportunistic pathogen, especially in immunocompromised hosts. The risk factors associated with acquiring *S. maltophilia* infections are well-known and include underlying malignancy (especially hematologic malignancy), organ transplantation, human immunodeficiency virus (HIV) infection, cystic fibrosis, prolonged hospitalization, intensive care unit (ICU) admission, mechanical ventilation, indwelling catheters (vascular, urinary, biliary), corticosteroid or immunosuppressant therapy, and recent antibiotics treatment (Al-Anazi and Al-Jasser, 2014). These risk factors reflect specific features of *S. maltophilia*, such as its ability to survive on almost any humid surface, its propensity to form biofilm and colonize humid surfaces, and its employment of several mechanisms that confer resistance to a number of antimicrobial agents.

*S. maltophilia* causes a wide range of infections including respiratory tract infections (RTI), blood stream infections (BSI) and, less commonly, skin and soft tissue infections (SSTI), bone and joint infections, biliary tract infections, urinary tract infections, endophthalmitis, endocarditis, and meningitis (Falagas et al., 2009a; Looney et al., 2009). The correlations between *S. maltophilia* infection and structural abnormalities with or without obstruction or procedural manipulation are well documented. Biliary tract infections caused by obstruction due to hepatobiliary neoplasms (Papadakis et al., 1995; Chang et al., 2014) or post-operative anastomotic strictures of the gastrointestinal tract (Perez et al., 2014) have been reported in patients with biliary *S. maltophilia* sepsis. Pleural infections caused by post-surgical/tube thoracostomy or fistula (broncho-/esophageal-/bilio-) (Lee et al., 2014), post-neurosurgical meningitis (Sood et al., 2013; Lai et al., 2014b), complicated urinary tract infections (Vartivarian et al., 1996), and obstructive lung cancer (Fujita et al., 1996; Vartivarian et al., 2000) have all been reported to create a milieu for *S. maltophilia* infection. In addition, although commonly perceived as nosocomial pathogens, community-acquired infections appear to be on the rise (Falagas et al., 2009a; Chang et al., 2014).

## Prevalence

There were few data before 1970 regarding the prevalence or clinical characteristics of *S. maltophilia* (previously *Pseudomonas maltophilia* or *Xanthomonas maltophilia*) because of its rarity and relative clinical insignificance. It was in the 1980s when *S. maltophilia* became more frequently reported as an emerging nosocomial pathogen (Jang et al., 1992; Victor et al., 1994), especially in patients with post-chemotherapy neutropenia (Kerr et al., 1990; Labarca et al., 2000) and in those with indwelling central venous catheters (CVC) (Victor et al., 1994; Lai et al., 2006; Chen et al., 2014). Beginning in the late 1990s worldwide surveillance programs and multi-center studies began to provide more comprehensive information about the pathogenicity of *S. maltophilia*. Of the global surveillance programs, the SENTRY Antimicrobial Surveillance Program initiated in 1997 and the Study for Monitoring Antimicrobial Resistance Trends (SMART) initiated in 2002 are the most well-known (Jean et al., 2015). A number of nationwide and antimicrobial agent-targeted projects were also launched during the late 1990s, including the Canadian Ward Surveillance Study (CANWARD), the Surveillance and Control of Pathogens of Epidemiologic Importance (SCOPE) study, the British Society for Antimicrobial Chemotherapy (BSAC) Resistance Surveillance Project, the Taiwan Surveillance of Antimicrobial Resistance (TSAR) study, and the Tigecycline Evaluation Surveillance Trial (TEST).

Despite the massive scale of these surveillance studies, there are still limited integrated data on the prevalence and susceptibility patterns of *S. maltophilia*. The heterogeneity among the studies stems from the diverse patient demographics, geographic differences, and the ratio of the isolates collected from different sources, making inter-literature comparison difficult. To add to the complexity, there are no worldwide guidelines on susceptibility testing methodology and breakpoints for *S. maltophilia* (Nicodemo et al., 2004; Hombach et al., 2012), which results in different or absence of susceptibility breakpoints for some antibiotics. The lack of universal references for evaluating resistance of *S. maltophilia* to antimicrobial agents leads to confusion and complications when interpreting clinical data.

Table 1 shows the prevalence rates of infection due to *S. maltophilia*, categorized by sources of infection, reported by worldwide and nationwide surveillance projects as well as multi-center studies. Specific patient groups such as the critically ill in intensive care units (ICUs) and the pediatric population are presented separately in Table 1. By comparing data gathered by large surveillance studies over time we can estimate longitudinal change in prevalence of *S. maltophilia* infection in the general population. The frequency of occurrence among isolates from all sources ranged from 0.8 to 1.4% in five SENTRY studies during 1997~2003 (Fluit et al., 2001a; Gales et al., 2001a; Sader et al., 2004; Sader and Jones, 2005; Fedler et al., 2006b). During 2007–2012, the CANWARD surveillance study (Zhanel et al., 2011, 2013; Walkty et al., 2014) and the SENTRY antimicrobial surveillance program (Farrell et al., 2010b; Sader et al., 2013) reported prevalence rates ranging from 1.3 to 1.68%. These data indicate that there is an increasing trend in infections due to *S. maltophilia* in the general population.

**Table 1:** **Prevalence of ***S. maltophilia*** in worldwide surveillance and multicenter studies**.

**Country^a^**	**Study^b^**	**Year**	**Number of isolates**	**Prevalence and/or ranking**	**References**
All regions^c^	SENTRY	1997–1999	Total isolates:70067 SM^d^ isolates: 842	Among: all pathogens: 1.2%	Gales et al., 2001a
EU	SENTRY	1997–1998	Total/SM isolates in: BSI^e^: 9194/82 RTI: 2052/54 SSTI: 2320/13 UTI: 2138/3	Among: all pathogens: 1.0% BSI: 0.89%, ranking: 19th RTI: 2.63%, ranking: 9th SSTI: 0.56%, ranking: 19th UTI: 0.14%, ranking: 25th	Fluit et al., 2001a
LA	SENTRY	1997–2001	Total isolates: 19547 SM isolates: 166	Among: all pathogens: 0.8%	Sader et al., 2004
All regions	SENTRY	1997–2001	NFGNB^f^ isolates: 18569 SM isolates: 1488	Among: NFGNB: 8%	Jones et al., 2003
All regions	SENTRY	1997–2003	Total isolates: 221084 NFGNB^f^ isolates: 25305 Uncommon NFGNB^g^: 3509 SM isolates: 2076	Among: all pathogens: 0.94% NFGNB: 8.20% Uncommon NFGNB: 59.16%	Sader and Jones, 2005
All regions	SENTRY	2001–2004	GNB isolates: 54731 NFGNB isolates: 13808 SM isolates: 1256	Among: GNB: 2.29% NFGNB: 9.10%	Gales et al., 2006
Canada	CANWARD	2007–2009	Total isolates: 18538 GNB isolates: 8949^h^ SM isolates: 245	Among: all pathogens: 1.3%, ranking: 17th GNB: 2.7%	Zhanel et al., 2011
AP	SENTRY	2008	Total isolates: 5759 SM isolates: 97	Among: all pathogens: 1.68%	Farrell et al., 2010b
Canada	CANWARD	2008	Total isolates: 5282 SM isolates: 57	Among: all pathogens: 1.1%, ranking: 17th	Zhanel et al., 2010
France	MTC	2008–2009	Total isolates: 46400 Uncommon NFGNB isolates: 158 SM isolates: 61	Among: all pathogens: 0.13% Uncommon NFGNB: 39%	Fihman et al., 2012
Canada	CANWARD	2007–2011	Total isolates: 27123 SM isolates: 378	Among: all pathogens: 1.4%, ranking 16th	Zhanel et al., 2013
All regions	SENTRY	2011	Total isolates: 22005 SM isolates: 362	Among: all pathogens: 1.6%	Sader et al., 2013
Canada	CANWARD	2011–2012	Total isolates: 6593 SM isolates: 104	Among: all pathogens: 1.6%	Walkty et al., 2014
**BSI**					
USA	SCOPE	1995–1996	NFGNB isolates: 270 SM isolates: 18	Among: NFGNB: 6.7%	Jones et al., 1997
USA, Canada	SENTRY	1997	Total isolates:5058 SM isolates: 40	Among: all pathogens: 0.8%, ranking: 15th In USA: 0.7%, in Canada: 1.1%	Pfaller et al., 1998
NA, LA	SENTRY	1997	Total isolates: 9519 GNB isolates: 4267 SM isolates: 69	Among: all pathogens: 0.7% GNB: 1.6%	Diekema et al., 1999
EU	SENTRY	1997–1998	Total isolates: 9194 SM isolates: 82	Among: all pathogens: 0.89%, ranking: 19th	Fluit et al., 2001a
All regions	SENTRY	1997–1999		Among all pathogens in: AP: 0.9%, Canada: 0.6% EU: 0.9%, LA: 0.8%, USA: 0.7%	Gales et al., 2001a
LA	SENTRY	1997–2000	NA	Among: all pathogens: 0.7% 1997: 0.9%, 1998: 0.8%, 1999: 0.6%, 2000: 0.3%	
LA	SENTRY	1997–2001	Total isolates: 9058 SM isolates: 86	Among: all pathogens: 0.95%	Sader et al., 2004
Worldwide	MTC	2000–2004	All isolates: 26474 SM isolates: 203	Among: all pathogens: 0.8%	Sader et al., 2005b
**RTI**					
NA	SENTRY	1997	Total isolates: 2757 SM isolates: 99	Among: all pathogens: 3.6%, ranking: 8th In USA: 3.5%, in Canada: 3.7%	Jones et al., 2000
LA	SENTRY	1997	Total isolates: 556 SM isolates: 13	Among: all pathogens: 2.3%, ranking: 8th	Sader et al., 1998
NA	SENTRY	1998	Total isolates: 2773 SM isolates: 114	Among: all pathogens: 4.1%, ranking: 8th In USA: 3.7%, in Canada: 5.9%	Mathai et al., 2001
EU	SENTRY	1997–1998	Total isolates: 2052 SM isolates: 54	Among: all pathogens: 2.63%, ranking: 9th	Fluit et al., 2001a
All regions	SENTRY	1997–1999		Among all pathogens in: AP: 2.8%, Canada: 5.2% EU: 3.2%, LA: 1.8%, USA: 3.3%	Gales et al., 2001a
LA	SENTRY	1997–2000	Total isolates: 2505 SM isolates: 41	Among: all pathogens: 1.6%	Gales et al., 2002
LA	SENTRY	1997–2001	Total isolates: 3346 SM isolates: 60	Among: all pathogens: 1.8%	Sader et al., 2004
NA	SENTRY	2000	SM isolates: 94	Among: all pathogens: 3.5%	Hoban et al., 2003
NA, LA, EU	SENTRY	2004–2008	Isolates from HABP and VABP^i^ Total cases: 31436	Regional incidence: all regions: 3.1% USA: 3.3%, LA: 2.3%, EU: 3.2%	Jones, 2010
Canada	CANWARD	2008	Total isolates: 1612 SM isolates: 42	Among: all pathogens: 2.6%, ranking: 9th	Zhanel et al., 2010
USA and EU	SENTRY	2009–2012	Total isolates: 12851 GNB isolates: 8201	Among all pathogens in: USA: 4.4%, ranking: 6th EU: 3.2%, ranking:9th GNB: 6.02%	Sader et al., 2014a
USA and EU	MTC	2012	Total isolates: 2968 SM isolates: 186	Among: all pathogens:6.3%	Farrell et al., 2014
**UTI**					
NA	SENTRY	1997	Total isolates: 1698 GNB isolates: 80% SM isolates: 6	Among: all pathogens: 0.35% GNB: 0.44%	Jones et al., 1999b
EU	SENTRY	1997–1998	Total isolates: 138 SM isolates: 3	Among: all pathogens: 0.14%, ranking: 25th	Fluit et al., 2001a
All regions	SENTRY	1997–1999		Among all pathogens in: AP: 0.2%, Canada: 0.0% EU: 0.2%, LA: 0.0%, USA: 0.3%	Gales et al., 2001a
LA	SENTRY	1997–2001	Total isolates: 1961 SM isolates: 0	Among: all pathogens: 0%	Sader et al., 2004
AP region	SMART^j^	2009–2010	Total GNB isolates: 1762	Among all GNB in: China: 1.3%, Thailand: 3.3%	Lu et al., 2012
USA	SMART	2009–2011	Total GNB isolates: 2135 SM isolates: 6	Among: all GNB: 0.28%	Bouchillon et al., 2013
**IAI**					
China	SMART	2002–2009	Total GNB isolates: 3420 SM isolates: 50	Among: all GNB: 1.5% NFGNB: ranking: 3rd	Yang et al., 2010
AP region	SMART	2003–2010	Total GNB isolates: 20710 NFGNB isolates: 2252 SM isolates: 204	Among: all GNB: 1.0% NFGNB: 9.1%	Liu et al., 2012
Taiwan	SMART	2006–2010	Total GNB isolates: 2417 SM isolates: 28	Among: all GNB: 1.2%	Lee et al., 2012
Africa and middle east	TEST	2007–2012	Total isolates of cSSSI^14^ and IAI from TEST: 1990 and 255 GNB isolates from IAI: 225 SM isolates rom IAI: 16	Among: all pathogens in IAI: 6.3% GNB in IAI: 7.3%	Renteria et al., 2014
**SSTI**					
NA	SENTRY	1997	Total isolates: 1562 SM isolates: 15	Among: all pathogens: 0.96%	Doern et al., 1999
EU	SENTRY	1997–1998	Total isolates: 2320 SM isolates: 13	Among: all pathogens: 0.56%, ranking: 19th	Fluit et al., 2001a
All regions	SENTRY	1997–1999		Among all pathogens in: USA: 1.0%, Canada: 1.1% AP: 0.1%, EU: 0.6%, LA: 0.4%,	Gales et al., 2001a
LA	SENTRY	1997–2001	Total isolates: 1780 SM isolates: 7	Among: all pathogens: 0.39%	Sader et al., 2004
**ICU**					
EU	SENTRY	1997–1998	Total isolates from ICU: 3981	Among: all pathogens: 1.6%, ranking: 14th BSI: 1.1%, ranking: 15th RTI: 3.0%, ranking: 8th UTI: 0.0%	Fluit et al., 2001b
LA	SENTRY	1997–2001	Total isolates: 19547 SM isolates: 166	Among all pathogens in: ICU: 2.77%	Sader et al., 2004
NA	SENTRY	2001	Total isolates from ICU: 1321 SM isolates: 40 Respiratory source: 89.0%	Among: all pathogens: 3.0%, ranking: 10th	Streit et al., 2004
NA, LA, EU, Asia-Australia area	MTC	2000–2004	Isolates from ICU patients Total isolates: 9093 SM isolates: 131	Among: all pathogens: 1.4%	Sader et al., 2005a
German	SARI	2003–2004	Isolates collected from 39 German ICUs Total isolates:28266 GNB isolates: 12234	Among all pathogens: Median percentage: 1.7%	Meyer et al., 2006
Canada	CAN-ICU	2005–2006	Isolates from ICU patients Total isolates:4180 SM isolates: 108	Among: all pathogens: 2.6%	Zhanel et al., 2008
Korea	MTC	2008–2009	Respiratory tract isolates from patient with HABP in ICUs Total isolates: 372 CRGNB isolates: 82 SM isolates: 10	Among: all pathogens: 2.7% CRGNB^k^: 11.6%	Kim et al., 2014
EU	MTC(27)9 countries	Published in 2011	Respiratory tract isolates from patient with HABP in ICUs Total isolates: 495 SM isolates: 13	Among: all pathogens: 2.6%	Magret et al., 2011
**PEDIATRIC POPULATION**					
NA	SENTRY	1998–2003	Total isolates: 59826 Total isolates from pediatric patients < 7 years: 4641 SM isolates: 166	Among: all pathogens in: all ages: 1.4%, pediatric: 1.2%, both ranking: 10th	Fedler et al., 2006b
NA, LA, EU	SENTRY	2004	Total isolates from pediatric patients ≤18 years: 3537 SM isolates: 53	Among: all pathogens: 1.5%, ranking: 15th all regions combined	Fedler et al., 2006a
**COMMUNITY-ACQUIRED**					
USA, Canada, LA	SENTRY	1997	BSI SM isolates: 69	CA^l^/N/unknown: 23/28/18 CA: 33.3%	Diekema et al., 1999
UK and Ireland	BSAC	2001–2006	BSI SM isolates: 165	C/N: 31/66 CA: 33%	Livermore et al., 2008
AP region	SMART	2003–2010	IAI SM isolates: 204	CA/N: 26/125 CA: 17.2%	Liu et al., 2012
France	MTC	2008–2009	All sources SM isolates: 61	CA/N: 9/29 CA: 23.7%	Fihman et al., 2012
Taiwan	SMART	2006–2010	IAI SM isolates: 28	CA/N^i^: 3/18 CA: 14.3%	Lee et al., 2012

a *NA, North America; LA, Latin America; EU, Europe; USA, the United States of America; UK, United Kingdom; AP, Asia-Pacific*.

b *SENTRY, The SENTRY Antimicrobial Surveillance Program; SMART, Study for Monitoring Antimicrobial Resistance Trends; CAN-ICU, The Canadian Intensive Care Unit Surveillance Study; CANWARD, The Canadian Ward Surveillance Study; SARI, Surveillance of Antibiotic Use and Bacterial Resistance in ICUs(German); BSAC, The British Society for Antimicrobial Chemotherapy Resistance Surveillance Project; TEST, Tigecycline Evaluation Surveillance Trial; TIST, Tigecycline In Vitro Surveillance in Taiwan; TSAR, Taiwan Surveillance of Antimicrobial Resistance; SCOPE, Surveillance and Control of Pathogens of Epidemiologic Importance (USA); MTC, multicenter studies*.

c *The SENTRY Antimicrobial Surveillance Program has monitored the predominant pathogens and antimicrobial resistance in 5 geographic regions: Asia-Pacific, Europe, Latin America, Canada, and the United States (Gales et al., 2001a)*.

d *SM, Stenotrophomonans maltophilia*.

e *BSI, bloodstream infection; RTI, respiratory tract infection; IAI, intra-abdominal infection; UTI, urinary tract infection; SSTI, skin and soft tissue infection*.

f NFGNB, non-fermentative Gram-negative bacilli

g *Uncommon NFGNB, Acinetobacter spp. and Pseudomonas aeruginosa excluded*.

h *Of the 18538 organisms collected, the 20 most common represented 16780 (90.5%) of the isolates and underwent susceptibility testing, which included 8949 (53.3%) Gram-negative bacilli*.

i *HABP, hospital-acquired bacterial pneumonia; VABP, ventilator-associated bacterial pneumonia*.

j *SMART is a global surveillance program that has monitored the in vitro susceptibility patterns of clinical Gram-negative bacilli to antimicrobial agents collected worldwide from intra-abdominal infections since 2002 and urinary tract infections since 2009 (Morrissey et al., 2013)*.

k *CRGNB, carbapenem-resistant Gram-negative bacteria*.

l *C, community-acquired (collected within 48 h of hospitalization); N, nosocomial (collected more than 48 h after hospitalization)*.

It has been observed in the general population (Gales et al., 2001a) and in ICUs (Fluit et al., 2001b) alike that *S. maltophilia* is most frequently associated with respiratory tract infections (RTIs), followed by bloodstream infections (BSIs), and, rarely, skin and soft tissue infections (SSTIs) and urinary tract infections (UTI) (Gales et al., 2001a). The prevalence of RTIs due to *S. maltophilia* is generally higher than that of other infections caused by that pathogen, but varies widely among countries and continents, ranging from 1.6 to 6.3% during the period 1997–2012 (Sader et al., 1998, 2004, 2014a; Jones et al., 2000; Fluit et al., 2001a; Gales et al., 2001a, 2002; Mathai et al., 2001; Hoban et al., 2003; Jones, 2010; Zhanel et al., 2010; Farrell et al., 2014). The United States has the most consecutive records regarding RTI isolates collected by the SENTRY program. Based on data from four SENTRY studies (Gales et al., 2001a; Hoban et al., 2003; Jones, 2010; Sader et al., 2014a), the prevalence rates increased from 3.3–3.5% during 1997–2004 to 4.4% during 2009–2012. During that 15-year period, *S. maltophilia* went from being the eighth to the sixth most common cause of RTI. In a large study on 2968 RTI isolates collected from 59 medical centers in the USA and 15 centers in European countries in 2012, 6.3% of the pathogens were *S. maltophilia* (Farrell et al., 2014). These observations suggest an increasing frequency of occurrence of respiratory tract infections due to *S. maltophilia*.

*S. maltophilia* is less frequently isolated from patients with BSIs, UTIs, or SSTIs than from patients with RTIs, with reported isolation rates ranging from 0.7 to 1.1% for BSIs (Jones et al., 1997; Pfaller et al., 1998; Diekema et al., 1999; Fluit et al., 2001a; Gales et al., 2001a; Sader et al., 2004, 2005b), 0–0.3% for UTIs (Pfaller et al., 1998; Jones et al., 1999b; Fluit et al., 2001a; Gales et al., 2001a; Sader et al., 2004, 2005b), and 0.39–0.96% for SSTIs (Diekema et al., 1999; Fluit et al., 2001a; Gales et al., 2001a; Sader et al., 2004). SMART studies have also shown that isolation of *S. maltophilia* from intra-abdominal infections (IAIs) is also fairly uncommon, with rates ranging from 1 to 1.7% (2002–2010) (Guembe et al., 2008; Yang et al., 2010; Lee et al., 2012; Liu et al., 2012). However, data from African and Middle Eastern countries collected as part of the Tigecycline Evaluation Surveillance Trial during 2007–2012 (Renteria et al., 2014) revealed an uncommonly high rate of isolation (6.3%) of *S. maltophilia* from patients with IAIs. In addition, the results from a SMART study surveying UTIs in the Asian-Pacific region during 2009–2010 disclosed higher rates of *S. maltophilia* isolated from patients with UTIs in China (1.3%) and Thailand (3.3%) than in other countries (Lu et al., 2012), although the rates were not as high as those in certain countries in Africa and the Middle East.

## Gram-negative bacilli (GNB) and non-fermenting gram-negative bacilli (NFGNB)

The worldwide rate of isolation of *S. maltophilia* among GNB pathogens ranges from 2.29 to 2.7% according to a SENTRY study (2001–2004) (Gales et al., 2006) and a CANWARD surveillance study (2007–2009) (Zhanel et al., 2011). In the US state of Texas, however, a study at the M. D. Anderson Cancer Center revealed an increasing trend in the ratio of *S. maltophilia* among GNB isolates obtained from cancer patients during 1986–2002 (from 2% in 1986 to 7% in 2002) (Safdar and Rolston, 2007).

Among NFGNB, *S. maltophilia* has been reported to be the third most commonly isolated pathogen after *Pseudomonas aeruginosa* and *Acinetobacter baumannii*. In a large survey conducted as a part of the SENTRY program, 221,084 GNB isolates were collected worldwide, including 25,305 (11.5%) NFGNB isolates, of which *Acinetobacter* spp. and *P. aeruginosa* accounted for the vast majority (87.7%). The remaining 3509 isolates were deemed unusual NFGNB species. Of them, *S. maltophilia* was the most frequently isolated (*n* = 2076, 59.16%) (Sader and Jones, 2005). A similar finding was reported in a prospective multi-center study involving nine teaching hospitals in France, in which *S. maltophilia* was the most commonly isolated NFGNB among all unusual NFGNB species (39%) (Fihman et al., 2012). Other surveillance studies, namely SCOPE (Jones et al., 1997), SENTRY (Jones et al., 2003; Gales et al., 2006), and SMART (Liu et al., 2012) showed a steady increase in isolation of *S. maltophilia* among all NFGNB pathogens during the period 1995–2010 (6.7% in 1995–1996, 8.0% in 1997–2001, and 9.1% in 2001–2010). These findings show that *S. maltophilia* is not an insignificant pathogen among disease-causing GNB and NFGNB species.

## Intensive care units, pediatric population, and community-acquired infections

As expected, the prevalence of infections due to *S. maltophilia* is higher in intensive care units (1.4–3.0%) than in the general population (Fluit et al., 2001b; Sader et al., 2004; Streit et al., 2004; Sader et al., 2005a; Meyer et al., 2006; Zhanel et al., 2008; Magret et al., 2011; Kim et al., 2014).

There is limited information on the worldwide prevalence of *S. maltophilia* infections in the general pediatric population. SENTRY studies conducted during 1998–2003 (Fedler et al., 2006b) and in 2004 (Fedler et al., 2006a) showed that the prevalence of infections due to *S. maltophilia* was 1.2% among children ≤ 7 years and 1.4% among children ≤ 18 years old. The rates are similar to those in the adult population. A comparison of two single-center studies in China and the USA revealed markedly different incidence rates of ventilator-associated pneumonia due to *S. maltophilia* among pediatric patients in ICUs. Ning et al. reported a rate of 20.3% among patients aged 2 months to 16 years in a pediatric ICU in China (Ning et al., 2013) whereas Arthur et al. found that the rate of infection due to *S. maltophilia* among infants aged 0–6 months in a cardiac ICU in the USA was only 0.8% (Arthur et al., 2015).

Several recent studies have shown that *S. maltophilia* is also an emerging opportunistic pathogen in community settings (Falagas et al., 2009a; Chang et al., 2014). Results of a worldwide SENTRY study in 1997 (Diekema et al., 1999) and the British Society for Antimicrobial Chemotherapy Resistance surveillance project conducted during 2001–2006 (Livermore et al., 2008) showed that 33.3 and 32%, respectively, of *S. maltophilia* isolates were collected within 48 h after admission (defined as community-acquired in these studies) from patients with bloodstream infections. The results from two recent SMART studies revealed that 14.3–17.2% of isolates from patients with community-acquired IAI (also defined by a 48-h time frame within admission) during 2003–2010 were *S. maltophilia* (Lee et al., 2012; Liu et al., 2012). Another recent study on the prevalence of community-acquired *S. maltophilia* BSI in Taiwan, which specifically divided the patients into three categories based on whether they had community-acquired (excluding patients hospitalized within 90 days before admission, cared in a nursing facility, etc.), healthcare-associated or hospital-acquired infections, reported that 17.6% of all community-acquired bloodstream infections were due to *S. maltophilia* (Chang et al., 2014). A similar study in France revealed that 23.7% of all community-acquired BSIs were due to *S. maltophilia* (Fihman et al., 2012). These studies show that community-acquired *S. maltophilia* infections are far less rare than previously thought.

## Risk factors of mortalty

A number of risk factors for death due to *S. maltophilia* infections have been reported. Paez et al. (Paez and Costa, 2008) reviewed the literature from 1985 to 2008 and found that BSI and pneumonia, shock, thrombocytopenia, and Acute Physiological Assessment and Chronic Health Evaluation (APACHE) score >15 are independent risk factors associated with outcome. In addition, underlying hematological malignancy and admission to ICU are independent risk factors for cancer patients. The impact of appropriate antimicrobial treatment and removal of CVC on mortality were concluded to require further clinical studies (Paez and Costa, 2008). The conclusion of the review corresponds to the aforementioned studies. Falagas et al. analyzed 15 articles for attributable mortality of *S. maltophilia* infections. Only four studies provided relevant data regarding inappropriate antibiotic treatment, and three out of the four studies found significantly higher mortality when compared with initial appropriate therapy (Falagas et al., 2009b).

## Antimicrobial susceptibility

There are limited antimicrobial options for infections due to *S. maltophilia* because of its extensive resistance to most antibiotics, including β-lactam antibiotics, cephalosporins, macrolides, aminoglycosides, and carbapenems. Interpretive breakpoints for susceptibility are available only for ticarcillin/clavulanate, ceftazidime, minocycline, levofloxacin, trimethoprim/sulfamethoxazole (TMP/SMX), and chloramphenicol (CLSI, 2015). Table 2 shows the rates of susceptibility of *S. maltophilia* to antimicrobial agents reported in the studies presented in Table 1. TMP/SMX is recognized as the drug of choice (Wang et al., 2014a). Resistance rates vary geographically but are generally less than 10% (Chung et al., 2013). However, high and various rates of resistance to TMP/SMX have been reported in patients with cancer (Vartivarian et al., 1994; Micozzi et al., 2000), cystic fibrosis (Saiman et al., 2002; Cantón et al., 2003; San Gabriel et al., 2004; Valenza et al., 2008), and in several countries, including Taiwan, Japan, Korea, Thailand, Spain, Mexico, Saudi Arabia, Turkey, and Canada (16–78.8%) (Valdezate et al., 2001; del Toro et al., 2002; Lai et al., 2004; Gülmez and Hasçelik, 2005; Memish et al., 2012; Wu et al., 2012; Rattanaumpawan et al., 2013; Rhee et al., 2013; Zhanel et al., 2013; Flores-Treviño et al., 2014; Hotta et al., 2014; Walkty et al., 2014; Wang et al., 2014a). In the present review, global surveillance data for the period 1997–2012 show that *S. maltophilia* continues to be highly susceptible to TMP/SMX (Table 2). Over that 15-year period, the susceptibility rates reported in worldwide SENRTY studies (Gales et al., 2001a; Jones et al., 2003; Gales et al., 2006; Sader et al., 2013, 2014a), a BSAC surveillance study (Livermore et al., 2008), and three large-scale multi-national studies (Sader et al., 2005b; Farrell et al., 2010a, 2014) ranged from 90 to 100%.

**Table 2:** **Susceptibility of ***S. maltophilia*** to various antimicrobial agents in worldwide surveillance and multicenter studies**.

**Country**	**Study**	**Year/subgroups**	**TMP/SMX^d^**	**LEVO^d^**	**CIP^d^**	**CAZ^d^**	**T/C^d^**	**MCN^d^**	**TGC^d^**	**TGCMIC_50∕90_**	**PB^d^**	**References**
NA, LA	SENTRY	1997		78.0	20.9							Jones et al., 1999a
All regions	SENTRY	1997–1999										Gales et al., 2001a
		AP	92		51	47	71					
		CAN	98		47	60	85					
		EU	90		79	72	86					
		LA	98		57	75	87					
		USA	95		55	67	90					
LA	SENTRY	1997–2001										Sader et al., 2004
		2001	98	98.6	55.7	54.3	45.7				59.2	
		4 years	97.1	88	43.4	57.8	56				NA	
		BSI	95.3	88.4	50	73.3	64					
		RTI	100	90	38.3	46.7	51.7					
All regions	SENTRY	1997–2001	92	86	32	54	86					Jones et al., 2003
All regions	SENTRY	1997–2003	95.3	86.1	30.9	52.9	55.7				67.6	Sader and Jones, 2005
All regions	SENTRY	2001–2004	97	86.9		52.4	47.6				72.4	Gales et al., 2006
NA, LA, EU, AP	MTC	2003–2008										Farrell et al., 2010a
		NA	97.6	82.5		51.0	46.1		94.5^a^	0.5/2	73.2	
		EU	98.9	83.7		45.2	42.7		95.3		72.6	
		AP	90.8	78.0		32.6	27.0		96.1		33.4	
		LA	95.5	91.3		48.8	36.7		96.5		76.4	
		ALL	96.0	83.4		44.8	39.1		95.5		64.6	
All regions	SENTRY	2011										Sader et al., 2013
		CLSI	94.5	77.3		36.7			92.3^a^	0.5/2		
		EUCAST	95	NA		NA			79.8			
**BSI**												
NA, LA	SENTRY	1997	90.9%	81.8	27.3	27.3	90.9					Diekema et al., 1999
NA	SENTRY	1998	73.9	87.0	52.2	65.2	55.7				73.9^c^	Gales et al., 2001b
All regions	MTC	2000–2004	98.0		29.6	56.9			93.1^a^	1/2	84.6	Sader et al., 2005b
UK and Ireland	BSAC	2001–2006	100						89^b^			Livermore et al., 2008

a *Tigecycline breakpoints of ≤2 μg/mL for susceptibility and ≥8 mg/L for resistance were used for comparison purposes only, as defined by the USFDA*.

b *Susceptibility to tigecycline at the breakpoint of 1 mg/L used for Enterobacteriaceae and Acinetobacter spp*.

c *Resistant strains with colistin and polymyxin B MICs of ≥4 mg/L*.

d *Antibiotics abbreviations: TMP/SMX, trimethoprim/sulfamethoxazole; LEVO, levofloxacin; CIP, ciprofloxacin; CAZ, ceftazidime; T/C, Ticarcillin/Clavulanate; PB, polymyxin B; TGC, tigecycline; MCN, minocycline*.

Ceftazidime and ticarcillin/clavulanate used to be the most effective among β-lactam drugs against *S. maltophilia*. However, recent studies have demonstrated resistance rates of more than 30% and a trend in decreasing susceptibility with ceftazidime (47–75% during 1997–1999 to 30.5–36.8% during 2009–2012) (Table 2) (Gales et al., 2001a; Farrell et al., 2010a; Sader et al., 2014b). The same is true for ticarcillin/clavulanate. During 1997–1998, the rates of susceptibility of *S. maltophilia* to that combination ranged from 71–90% but dropped to 27–46.1% during 2003–2008.

New fluoroquinolones exhibit better potency against *S. maltophilia* than ceftazidime or ticarcillin/clavulanate and have become reasonable alternatives. Nonetheless, a comparison of data from worldwide SENTRY studies reveals a decrease in sensitivity of *S. maltophilia* to levofloxacin, from 83.4% during the period 2003–2008 (Farrell et al., 2010a) to 77.3% in 2011 (Sader et al., 2013). Low susceptibility rates ranging from 64–69.6% have also been reported in Canada (Zhanel et al., 2013), China (Yang et al., 2010; Tan et al., 2014), and Korea (Chung et al., 2013). Few multi-center studies have investigated the efficacy of fluoroquinolones against *S. maltophilia* in patients with UTIs. In a SMART study conducted in the Asia-Pacific region, isolates of *S. maltophilia* from patients with UTIs showed exceptionally high rates of resistance to levofloxacin (33.3%) (Lu et al., 2012). Two recent reports showed low MIC_50_ (minimum inhibitory concentration)values (0.5 mg/L and 0.5 mg/L) and low MIC_90_ values (8 and 4 mg/L) for moxifloxacin against *S. maltophilia* (Zhanel et al., 2008; Chung et al., 2013), indicating that moxifloxacin could be considered an effective alternative. Data from a number of studies demonstrate that ciprofloxacin has poor activity against *S. maltophilia*, with susceptibility rates averaging lower than 50% (Table 2).

Minocycline, doxycycline, and tigecycline have consistently displayed good potency against *S. maltophilia* in studies with various time periods, sources of specimens, and geographic regions (Sader et al., 2005b, 2013, 2014b; Gales et al., 2008; Chen et al., 2012; Wu et al., 2012; Chung et al., 2013). A TSAR surveillance study conducted in Taiwan tested 377 isolates of *S. maltophilia* obtained over a 10-year period (1998–2008) and revealed low MIC_50_ (0.25 mg/L) and MIC_90_ values (1 mg/L) for tigecycline (Wu et al., 2012). Similar results were demonstrated in several large-scale worldwide surveillance studies as well. A recent SENTRY study conducted during 2009–2012 (494 isolates) (Sader et al., 2014a) revealed a susceptibility of 96% and a recent TEST study conducted during 2007–2012 (2245 isolates) (Renteria et al., 2014) demonstrated low MIC_50_ (0.25 mg/L) and MIC_90_ (1 mg/L) values.

## Molecular mechanisms in antimicrobial resistance

*S. maltophilia* has several molecular mechanisms contributing to its extensive antimicrobial resistance. The mechanisms are summarized in Table 3. Detailed descriptions of the major mechanisms are elaborated as follows.

**Table 3:** **Molecular mechanisms of antimicrobial resistance in ***S. maltophilia*****.

**Mechanisms**	**Associated determinants**	**Related antimicrobial resistance**
β-lactamases 1. L1, L2 (chromosomally and plasmid encoded) 2. TEM-2 (on a Tn*1*-like transposon)	*ampR*-dependent (involving *ampR, ampN-ampG* operon, *ampD_*I*_ and mrcA*)	β-lactamases
Class 1 integrons and IS*CR* elements	*sul1, sul2, dfrA*	TMP/SMX
Multidrug efflux pump	**RND family**: SmeABC, SmeDEF, SmeGH^*^, SmeIJK, SmeMN^*^, SmeOP, SmeVWX, and SmeYZ **ABC family**: SmrA, MacABCsm **MFS family:** EmrCABsm	Summarized in Table 4
Qnr	Sm*qnr*	Quinolones and tetracycline
Antibiotic-modifying enzymes	AAC(6′)-Iz, APH(3′)-IIc, AAC(6′)-Iak	Aminoglycoside
Lipopolysaccharide (LPS)	SpgM (phosphoglucomutase)	Aminoglycosides, polymyxin B, ticarcillin/clavulanic acid and piperacillin/tazobactam
Mutations of bacterial topoisomerase and gyrase genes		
Reduction in outer membrane permeability		

* *not yet characterized*.

### β-Lactamases

*S. maltophilia* has two chromosomal-mediated inducible β-lactamases, namely L1 and L2. L1 is a molecular class B Zn^2+^-dependent metallo-β-lactamase and L2 is a molecular class A clavulanic acid-sensitive cephalosporinase. The L1 and L2 β-lactamases are simultaneously regulated by AmpR, a transcriptional regulator in the L2 upstream region (Okazaki and Avison, 2008). The *ampR*-L2 module is homologous to the *ampR-ampC* systems, which are widely distributed in some members of the family *Enterobacteriaceae* and in *P. aeruginosa* (Lodge et al., 1990). The regulation of chromosomal *ampR-ampC* systems has been well studied in *Citrobacter freundii*, where the AmpC β-lactamase induction is linked to peptidoglycan recycling and involves several regulatory genes, such as as *ampR, ampG*, and *ampD* (Lindberg et al., 1985). A similar induction mechanism was proposed for the *ampR-ampC* and the *ampR*-L2 modules (Okazaki and Avison, 2008). But unlike *P. aeruginosa*, the permease system in *S. maltophilia* requires an intact *ampN*-*ampG* operon for the induction of β-lactamase (Huang et al., 2010). Two *ampD* homologs, *ampD*_*I*_ and *ampD*_*II*_, were found in *S. maltophilia*, but only *ampD*_*I*_appears to be relevant to the regulation of β-lactamase (Yang et al., 2009).

Penicillin-binding proteins (PBPs) participate in peptidoglycan biosynthesis and the inactivation of PBP4 in *P. aeruginosa* has been shown to confer AmpC overexpression and β-lactam resistance (Moya et al., 2009). The inactivation of a putative PBP1a gene, *mrcA*, recently was found to cause basal-level L1/L2 β-lactamase hyperproduction in *S. maltophilia* KJ. The inactivation of *mrcA* only affects basal L1/L2 production β-lactamase, which is *ampR*- and *ampN-ampG*-dependent, and does not augment their induction (Lin et al., 2011). The universality of disruption of *ampD*_*I*_ or *mrcA* in β-lactamase-hyperproducing *S. maltophilia* mutants and clinical isolates has been proved by the existence of wild-type *ampD*_*I*_ and *mrcA* genes. The result implicates mutation of at least one additional gene in this phenotype (Talfan et al., 2013).

### Efflux pumps

Efflux pumps in microorganisms mediate the extrusion of drugs and are classified into five families, namely the resistance-nodulation-cell-division (RND) family, the major facilitator superfamily (MFS), the small multidrug resistance (SMR) family, the ATP binding cassette (ABC) family, and the multidrug and toxic compound extrusion (MATE) family (Putman et al., 2000). Two ABC-type (SmrA, MacABCsm), one MFS-type (EmrCABsm), a fusaric acid extrusion efflux pump (FuaABC), and six out of the eight postulated RND-type efflux systems have been characterized in *S. maltophilia* (Alonso and Martinez, 2000; Li et al., 2002; Crossman et al., 2008; Al-Hamad et al., 2009; Chen et al., 2011; Hu et al., 2012; Gould et al., 2013; Huang et al., 2013a; Lin et al., 2014a,b). The six characterized RND-type efflux pumps in the *S. maltophilia* genome are SmeABC, SmeEF, SmeIJK, SmeOP, SmeVWX, and SmeYZ (including SmeGH and SmeMN). Table 4 provides a summary of antimicrobial resistance associated with the abovementioned efflux pumps.

**Table 4:** **Genetic determinants of efflux pumps**.

**Efflux pumps**	**Associated antibiotic resistance**
**RND FAMILY**	
SmeABC	Quinolones, ß-lactams and aminoglycosides
SmeDEF	Quinolones, tetracyclines, macrolides, chloramphenicol, novobiocin and trimethoprim/sulfamethoxazole
SmeIJK	Ciproxin, levofloxacin, tetracycline and minocycline
SmeOP-TolCsm	Trimethoprim/sulfamethoxazole, aminoglycosides, macrolides, doxycycline, chloramphenicol, and nalidixic acid
SmeVWX	Quinolones, chloramphenicol and tetracyclines
SmeYZ	Trimethoprim/sulfamethoxazole and aminoglycosides
**ABC FAMILY**	
SmrA	Fluoroquinolones and tetracycline
MacABCsm	Aminoglycosides, macrolides and polymyxins
**MFS FAMILY**	
EmrCABsm	Nalidixic acid and erythromycin
**FUSARIC ACID TRIPARTITE EFFLUX PUMP**	
FuaABC	fusaric acid

#### SmeABC

The overexpression of *smeABC* genes confers resistance to aminoglycosides, β-lactams, and fluoroquinolones. SmeC was identified to function independently of SmeAB, while deletions in *smeC* but not *smeB* compromised the antimicrobial resistance (Li et al., 2002).

#### SmeDEF

SmeDEF is a complex formed by an efflux pump located on the inner membrane (SmeE), an outer membrane protein (SmeF) and a periplasmic membrane fusion protein (SmeD). It is involved in resistance to quinolones, tetracyclines, macrolides, chloramphenicol and novobiocin (Alonso and Martinez, 2000). Expression of the *smeDEF* operon is regulated by the SmeT repressor (Hernandez et al., 2009). A recent study showed that the SmeDEF efflux pump is associated with plant root colonization by *S. maltophilia*, and that deletion of the *smeE* gene impairs this function (García-León et al., 2014a).

#### SmeVWX

The SmeVWX pump, encoded by a five-gene operon (*smeU1, smeV, smeW, smeU2*, and *smeX*), was identified and characterized in a multidrug-resistant mutant of *S. maltophilia* KJ. Overexpression of the SmeVWX pump resulted in increased resistance to chloramphenicol, quinolones, and tetracyclines but increased aminoglycoside susceptibility (Chen et al., 2011).

#### SmeYZ and SmeJK

The *smeZ, smeJ*, and *smeK* genes were identified in *S. maltophilia* K M5, a selected mutant derivative. SmeZ contributes to elevated aminoglycoside MICs. SmeJ and SmeK jointly elevate tetracycline, minocycline, and ciprofloxacin MICs and confer resistance to levofloxacin (Gould et al., 2013). In addition to drug extrusion, the SmeIJK pump has been reported to play a physiologic role in the maintenance of cell membrane integrity (Huang et al., 2014). A recent study further elucidated the physiologic significance of the SmeYZ pump and demonstrated its correlation with virulence-related functions, including swimming, flagella formation, oxidative stress susceptibility, biofilm formation, and protease secretion (Lin et al., 2015).

#### SmeOP

A *pcm-tolCsm* operon was recently verified in *S. maltophilia* KJ2. The *tolCsm* gene is involved in the resistance of several antimicrobial agents, including aminoglycosides, macrolides, β-lactams, chloramphenicol, nalidixic acid, doxycycline and TMP/SMX. The deletion of *pcm* was shown to result in decreased expression of *tolCsm*, which compromised the pathogen's resistance to amikacin and gentamicin (Huang et al., 2013b). A very recent study characterized a five-gene cluster efflux pump (*tolCSm-pcm-smeRo-smeO-smeP*) in *S. maltophilia*. The study showed that SmeOP requires TolCSm for efflux pump function and suggested that TolCSm is the cognate outer membrane protein (OMP) for the SmeOP pump. The SmeOP-TolCSm efflux pump was shown to be associated with resistance to nalidixic acid, doxycycline, amikacin, gentamicin, erythromycin, and leucomycin (Lin et al., 2014a).

#### ABC family: SmrA and MacABCsm

SmrA, the first ABC-type efflux pump identified in *S. maltophilia*, has been shown to confer resistance to fluoroquinolones and tetracycline (Al-Hamad et al., 2009). The MacABCsm efflux pump in *S. maltophilia* was recently shown to confer intrinsic resistance to antimicrobials (aminoglycosides, macrolides, and polymyxins) and to play an important role in regulating oxidative and envelope stress tolerance and biofilm formation (Lin et al., 2014b).

#### MFS family: EmrCABsm

Only one MFS-type efflux pump, EmrCABsm, has been characterized so far. It is involved in the extrusion of hydrophobic compounds, including the antibiotics nalidixic acid and erythromycin (Huang et al., 2013a).

#### Fusaric acid tripartite efflux pump

A novel tripartite fusaric acid efflux pump was found in *S. maltophilia*, namely FuaABC, which may constitute a new subfamily of the tripartite efflux pump. The *fuaABC* operon was demonstrated to be induced by fusaric acid and to contribute to fusaric acid resistance when overexpressed (Hu et al., 2012).

### Trimethoprim/sulfamethoxazole (TMP/SMX) resistance mechanisms

The *sul1* gene carried by class 1 integrons and the *sul2* gene, which is linked to insertion sequence common region (ISCR) elements, are known to be responsible for resistance to TMP/SMX in *S. maltophilia* (Barbolla et al., 2004; Toleman et al., 2007; Chung et al., 2015). The *dfrA* gene cassettes, which are located in class 1 integrons and encode for the dihydrofolate reductase enzyme, have also been reported to confer high-level resistance to TMP/SMX (Hu et al., 2011). Moreover, SmeDEF, TolCsm, and SmeYZ efflux pumps have recently been reported to be associated with TMP/SMX resistance (Huang et al., 2013b; Lin et al., 2015; Sánchez and Martínez, 2015).

### Quinolones resistance mechanisms

Two mechanisms are associated with resistance of *S. maltophilia* to quinolones: efflux pumps and a chromosomally encoded *qnr* gene (Sm*qnr*) that protects both gyrase and topoisomerase IV from quinolones (Sanchez et al., 2009). Unlike other bacteria, clinical isolates of quinolone-resistant *S. maltophilia* do not present mutations in topoisomerases (Valdezate et al., 2005). To date, the characterized genetic determinants involving resistance to quinolones are *smeDEF, smeIJK, smeABC, smeVWX*, and Sm*qnr* genes, among which *smeDEF* and Sm*qnr* are the best-described. Sm*qnr* belongs to the *qnr* family. It confers low-level resistance and contributes to intrinsic resistance to quinolones in *S. maltophilia* (Sánchez et al., 2008; Shimizu et al., 2008; Sánchez and Martínez, 2010). García-León et al. further elucidated the interplay between intrinsic and acquired resistance to quinolones in *S. maltophilia*. Their study demonstrated that the capacity to develop mutation-driven antibiotic resistance is highly dependent on the intrinsic resistome. Their findings indicate that the most prevalent cause of acquired quinolone resistance in *S. maltophilia* is the overproduction of multidrug efflux pumps, among which SmeDEF efflux pump plays the most important role (García-León et al., 2014b). In addition, a more recent report by García-León et al. confirmed that overexpression of *smeVWX* in clinical isolates of *S. maltophilia* is associated with high-level quinolone resistance (García-León et al., 2015).

### Aminoglycosides resistance mechanisms

The aminoglycoside-resistant mechanisms in *S. maltophilia* primarily involve aminoglycoside-modifying enzymes and efflux pumps. The reported enzymes to date include AAC(6′)-Iz (an aminoglycoside acetyltransferase) (Li et al., 2003), APH(3′)-IIc (an aminoglycoside phosphotransferase) (Okazaki and Avison, 2007) and a novel AAC(6′)-Iak, which was recently identified in a MDR strain from Nepal (Tada et al., 2014). Efflux pumps including SmeABC, SmeYZ, SmeOP-TolCsm, and MacABCsm are associated with resistance as described above.

## Antimicrobial treatment studies

Trimethoprim/sulfamethoxazole (TMP/SMX) remains the most effective antimicrobial agent against *S. maltophilia*, with an overall susceptibility rate higher than 90% (Falagas et al., 2008). A recent study investigated the efficacy of sulfametrole/trimethoprim, an alternative sulphonamide/trimethoprim combination available in several Europe countries against non-fermenters (40 *S. maltophilia* included) and found that the activity of the alternative combination was similar to that of TMP/SMX (Livermore et al., 2014). Other common options include ceftazidime, ticarcillin-clavulanate, fluoroquinolones, and tetracyclines such as tigecycline, minocycline, and doxycyclines. As previously mentioned, resistance rates to ceftazidime and ticarcillin-clavulanate are high and rising and are, therefore, unreliable choices. Fluoroquinolones are now popular alternatives because of their less prominent side effects compared to TMP/SMX and their greater potency compared to β-lactams.

### Fluoroquinolones

Fluoroquinolones (FQs) are commonly used to treat infections due to *S. maltophilia* (Nicodemo and Paez, 2007). However, their overuse worldwide has resulted in higher resistance rates in many kinds of pathogenic bacteria, including *S. maltophilia* (Chang et al., 2014; Pien et al., 2015).

To evaluate the effectiveness of FQs in this era of high FQ resistance, a retrospective study published in 2014 compared the outcomes of patients with *S. maltophilia* infections treated with TMP/SMX and those of patients treated with FQs monotherapy (Wang et al., 2014b). A total of 38 adults received TMP/SMX and 63 adults received FQs (levofloxacin *n* = 48 and ciprofloxacin *n* = 15). The overall microbiological cure rate was 63% (65% in the TMP/SMX group and 62% in the FQ group), and the overall clinical success rate was 55% (61% in the TMP/SXT group and 52% in the FQ group). The antibiotic regimens were equally effective in both groups. Another retrospective study compared the effectiveness of TMP/SMX (*n* = 51) with that of levofloxacin (*n* = 35) in treating *S. maltophilia* bacteremia and revealed no significant differences in treatment outcome between the two groups, including 30-day mortality, length of hospital day, and antibiotic withdrawal (Cho et al., 2014). However, the rate of adverse events was significantly lower in the levofloxacin group (0%) than in the TMP/SXT group (23.5%, *p* = 0.001).

Several new quinolones have been developed and some of them have recently been approved for clinical application, including nemonoxacin (Huang et al., 2015) and delafloxacin (Bassetti et al., 2015). Oral nemonoxacin, a novel nonfluorinated quinolone antibiotic, has been shown to have good activity against Gram-positive bacilli, such as methicillin-resistant *Staphylococcus aureus* (Huang et al., 2015). However, *in vitro* susceptibility assays on 32 clinical isolates of *S. maltophilia* revealed high MIC_90_ (32 mg/L) and MIC_50_ (8 mg/L) values (Lai et al., 2014a). Regarding delafloxacin, a phase II study published in 2009 that compared two doses of delafloxacin to tigecycline in adults with complicated skin and skin structure infections found that only one patient was infected with *S. maltophilia* and was treated successfully with delafloxacin (O'Riordan et al., 2015). More *in vivo* studies are needed to better understand the effectiveness of these new quinolones in treating *S. maltophilia* infections.

### Tetracyclines

Tetracyclines such as tigecycline, minocycline, and doxycycline are some of the most active antimicrobial agents against *S. maltophilia* other than TMP/SMX, even in the cystic fibrosis population (Cantón et al., 2003; San Gabriel et al., 2004; Gülmez et al., 2010; Milne and Gould, 2012; Castanheira et al., 2014). The results agree with our aforementioned observation that these antibiotics consistently exhibited good activity against *S. maltophilia* in global surveillance studies (Table 2).

Tigecycline, a derivative of minocycline, has broad-spectrum antimicrobial activity (Stein and Babinchak, 2013) and is an alternative agent against *S. maltophilia* infections. A recent study from Brazil showed that the MIC_50_ and MIC_90_ values of tigecyline for *S. maltophilia* isolates, including isolates resistant to levofloxacin and/or TMP/SMX harboring *sul-1, sul-2*, and *qnrMR*, were 1 and 4 μg/ml, respectively (Rizek et al., 2015). However, tigecycline has great bio-distribution after intravenous injection, which leads to lower serum drug levels. So, there are concerns about its efficacy for the treatment of bacteremia due to *S. maltophilia* (Stein and Babinchak, 2013). In a recent study, a high dose of tigecycline was effective at treating *S. maltophilia* bacteremia (Wu and Shao, 2014), although in another study, high-dose tigecycline treatment was associated with significant adverse effects (Falagas et al., 2014). In addition, a 3-year clinical therapeutic study that compared the effectiveness of TMP/SMX and tigecycline in treating nosocomial *S. maltophilia* infections revealed no significant differences in mortality or clinical response rates between the two regimens. Clinical improvement rates on the 14th day were 69.2% in the TMP/SMX group and 68.4% in the tigecycline group (*P* = 0.954) and mortality rates on the 30th day were 30.8% in the TMP/SMX group and 21.1% in the tigecycline group (*P* = 0.517) (Tekce et al., 2012). Therefore, tigecycline might be an alternative for patients who are unable to tolerate TMP/SMX. In addition to monotherapy, a combination regimen with tigecycline might be a better option for severe infections, especially for nosocomial infections (Samonis et al., 2012).

In a recent large collection of resistant organisms from the SENTRY program during 2007–2011 (1706 *S. maltophilia* included), minocycline was shown to be significantly more active than other tetracyclines against *S. maltophilia*. The rate of susceptibility of *S. maltophilia* to minocycline exceeded 97% across all geographic regions, and the potency was 2-fold higher than doxycycline (MIC_50/90_: 0.5/2 μg/mL) (Castanheira et al., 2014). A study evaluated 53 multidrug resistant isolates of *S. maltophilia*, including 48 that were resistant to levofloxacin and/or TMP/SMX, and found that minocycline exhibited excellent activity against *S. maltophilia*. However, the clinical experience is still anecdotal (Rizek et al., 2015). A patient with pneumonia was reportedly successfully treated with minocycline (Irifune et al., 1994), and the combination of minocycline with TMP and ticarcillin/clavulanate has been suggested to be effective (Vartivarian et al., 1994).

### Polymyxins and fosfomycin

Polymyxins and fosfomycin are being reconsidered as alternatives or “last-resort” options because of the increasing emergence of multidrug-resistant organisms. Unfortunately, interpreting the susceptibility rates of *S. maltophilia* to polymyxins is problematic because of the discordance between different testing methods (Nicodemo et al., 2004; Gülmez et al., 2010; Moskowitz et al., 2010; Betts et al., 2014). A SENTRY surveillance program study conducted during 2001–2004 assessed the antimicrobial activity of polymyxin B among 54731 isolates of GNB and 1256 isolates of *S. maltophilia* and found that 72.4% of the *S. maltophilia* isolates were susceptible to polymyxin B (MIC_50_ and MIC_90_ values, 1 and 8 mg/L, respectively) (Gales et al., 2006). Colistin (polymyxin E) appears to have a considerable *in vitro* activity against *S. maltophilia* (83–88%) (Falagas and Kasiakou, 2005). In addition, synergy with rifampin, TMP/SMX, and doxycycline has been shown (Giamarellos-Bourboulis et al., 2002; Gülmez et al., 2010). Two studies by the SENTRY program in the year 2011 (globally) and during 2009–2012 (USA and Europe) that collected 362 and 494 isolates of *S. maltophilia*, respectively, reported very different rates of susceptibility to colistin (98.5%, Sader et al., 2013 and 38.7–49.7%, Sader et al., 2014a). In a recent study that collected 641 isolates of *S. maltophilia* in a university hospital in Argentina (Rodríguez et al., 2014), Rodríguez et al. showed that colistin resistance increased from 8% in 1996 to 45% in 2013 and found that the increase correlated with a marked increase (11.4-fold) in colistin consumption during the study period. Fosfomycin has been shown in several reports to be an inappropriate treatment option because of its poor activity and high MICs against *S. maltophilia* (Macleod et al., 2009; Khan et al., 2014; Rizek et al., 2015).

### Combination therapy

Owing to the impressive array of antimicrobial resistance mechanisms of *S. maltophilia*, various combinations of antimicrobial agents have been surveyed in order to overcome resistance or to attain synergism (Table 5). Combinations of two or three agents with good susceptibility results such as TMP/SMX, ceftazidime, ticarcillin/clavulanate, and aminoglycosides have demonstrated synergistic effects to different degrees in prior studies. In more recent studies, combinations of TMP/SMX or β-lactam/β-lactam inhibitors with new or old antibiotics such as tigecycline, fluoroquinolones, televancin (Hornsey et al., 2012), rifampin (Betts et al., 2014), and aerosolized colistin have been investigated; they demonstrated various extents of synergism and the ability to maintain effectivity in biofilm. TMP/SMX plus ceftazidime plus levofloxacin has been shown to be effective in treatment of meningitis (Correia et al., 2014) and intrabiliary infusion of colistin plus parenteral fosfomycin with tigecycline was reported to be effective at treating complicated biliary tract infection (Perez et al., 2014). Several combinations of novel agents are currently under investigation, including a β-lactam and dual β-lactamase inhibitor combination (Page et al., 2011) and MD3 (a novel synthetic inhibitor of peptidases) plus colistin (Personne et al., 2014). It is important to mention that *in vitro* synergy attained by combination should be further correlated with clinical outcomes.

**Table 5:** **Combinations of antibiotics that demonstrate synergism and nebulized antimicrobial agents**.

**Year**	**Combinations**	**References**
**COMBINATIONS**		
1974	TMP/SMX + colistin	Nord et al., 1974
1979	TMP/SMX + carbenicillin	Felegie et al., 1979
1980	Gentamicin + carbenicillin + rifampin TMP/SMX + carbenicillin + rifampin	Yu et al., 1980
1983	Gentamicin + carbenicillin + rifampicin TMP/SMX + carbenicillin + rifampicin	Berenbaum et al., 1983
1988	Ciprofloxacin + ceftazidime	Chow et al., 1988
1995	Ticarcillin/clavulanate + TMP/SMX Ticarcillin/clavulanate + ciprofloxacin	Poulos et al., 1995
1998	Levofloxacin + various beta-lactams	Visalli et al., 1998
2001	Ticarcillin/clavulanate + aztreonam	Krueger et al., 2001
2002	Azithromycin + TMP/SMX	Saiman et al., 2002
2009	Tigecycline + amikacin Tigecycline + TMP/SMX	Entenza and Moreillon, 2009
2010	TMP/SMX + ticarcillin/clavulanate TMP/SMX + ceftazidime	Gülmez et al., 2010
2012	Ticarcillin/clavulanate + aztreonam: most synergic combination Ticarcillin/clavulanate + colistin Ticarcillin/clavulanate + levofloxacin	Milne and Gould, 2012
2012	Telavancin + colistin: marked synergy	Hornsey et al., 2012
2013	Tigecycline + colistin: best result	Church et al., 2013
2013^a^	Ceftazidime + colistin^200^ Levofloxacin^100^ + ticarcillin/clavulanate Colistin^200^ + TMP/SMX	Wu et al., 2013
2013	TMP/SMX + ticarcillin/clavulanate: most synergistic combination	Chung et al., 2013
2014	Ceftazidime + TMP/SMX Ceftazidime + levofloxacin More effective than the combination of TMP/SMX + levofloxacin	Hu et al., 2014
2014	Colistin + rifampin: reliably bactericidal Colistin + tigecycline	Betts et al., 2014
**NOVEL COMBINATIONS**		
2011	BAL30376: β-lactam and dual β-lactamase inhibitor combination	Page et al., 2011
2014	MD3 + colistin MD3: a novel synthetic inhibitor of SPases (bacterial type I signal peptidases)	Personne et al., 2014
**NEBULIZED ANTIMICROBIAL AGENTS**		
2010	Doxycycline + aerosolized colistin	Wood et al., 2010; Harthan and Heger, 2013
2010	Levofloxacin	King et al., 2010
2015	Tobramycin inhalation powder	Ratjen et al., 2015

*TMP/SMX, trimethoprim/sulfamethoxazole*.

a *The Top 3 effective combinations when S. maltophilia isolates grown as a biofilm. Colistin and levofloxacin were tested at high concentrations (200 and 100 mg/L, respectively), corresponding to the level achievable in sputum by aerosolization*.

### Nebulized antimicrobial agents

Nebulization of antimicrobials results in high concentrations in the respiratory tract and is associated with low toxicity because this method of delivery results in limited systemic absorption (Table 5). These characteristics are especially important for patients with cystic fibrosis, who are prone to frequent infection and colonization of multidrug resistant pathogens including *S. maltophilia*. Wood et al. reported a case of recurrent ventilator-associated pneumonia successfully treated with aerosolized colistin and doxycycline (Wood et al., 2010). King et al. surveyed the *in vitro* pharmacodynamics of aerosolized levofloxacin and suggested that the high concentrations of levofloxacin achieved in the lung by aerosol delivery may be useful for the treatment of patients with cystic fibrosis (King et al., 2010). When *S. maltophilia* isolates were grown as a biofilm, the top 3 most effective antibiotic combinations included high-dose levofloxacin or colistin delivered at doses achievable by aerosolization plus a β-lactam or TMP/SMX (Table 5) (Wu et al., 2013). The potentials of other antibiotics to be nebulized to achieve high drug levels in airway have been investigated in order to overcome the high MICs that cannot be conquered when the agents are administered systemically. A device (Podhaler device) that delivers new inhalational tobramycin (tobramycin inhalation powder, TIP) and attains high drug levels to the lung may be able to exceed current high MICs of tobramycin in *S. maltophilia* (Ratjen et al., 2015). Waters suggested a potential role of inhaled aztreonam lysine in the treatment of *S. maltophilia* pulmonary infection because of its resistance to the L1 β-lactamase produced by *S. maltophilia* and the ability to achieve high drug levels in respiratory secretions (approximately 1000-fold higher than the corresponding plasma concentration) (Waters, 2012). The antibacterial activity of a novel inhaled combination of fosfomycin and tobramycin (FTI) was investigated in patients with bronchiectasis. However, FTI demonstrated relatively poor activity against *S. maltophilia* (Macleod et al., 2009).

## Conclusions

Worldwide, multi-institutional studies confirm that *S. maltophilia* is an emerging multi-drug resistant opportunistic pathogen in hospital and community settings, especially among immunocompromised hosts. TMP/SMX remains the most effective antimicrobial agent in the general population. Drugs with historically good susceptibility results include ceftazidime, ticarcillin-clavulanate, and fluoroquinolones; however, a number of studies show an alarming trend in resistance to those agents. Tetracyclines such as tigecycline, minocycline, and doxycycline are also effective agents and consistently display good activity against *S. maltophilia* in various geographic regions and across different time periods. Combination therapies, novel agents, and aerosolized forms of antimicrobial drugs are currently being tested for their ability to treat infections caused by this multi-drug resistant organism. In addition, recent advances in molecular methods have identified various new mechanisms contributing to drug resistance, which hopefully will lead to future breakthroughs in the treatment of infections due to *S. maltophilia*.

### Conflict of interest statement

The authors declare that the research was conducted in the absence of any commercial or financial relationships that could be construed as a potential conflict of interest.

## References

[B1] Al-Anazi K. A. Al-Jasser A. M. (2014). Infections caused by *Stenotrophomonas maltophilia* in recipients of hematopoietic stem cell transplantation. Front. Oncol. 4:232 10.3389/fonc.2014.00232 PMC4142553 25202682

[B2] Al-Hamad A. Upton M. Burnie J. (2009). Molecular cloning and characterization of SmrA, a novel ABC multidrug efflux pump from *Stenotrophomonas maltophilia*. J. Antimicrob. Chemother. 64, 731–734. 10.1093/jac/dkp271 19643774

[B3] Alonso A. Martínez J. L. (2000). Cloning and characterization of *SmeDEF*, a novel multidrug efflux pump from *Stenotrophomonas maltophilia*. Antimicrob. Agents Chemother. 44, 3079–3086. 10.1128/AAC.44.11.3079-3086.2000 11036026 PMC101606

[B4] Arthur C. Tang X. Romero J. R. Gossett J. G. Harik N. Prodhan P. (2015). *Stenotrophomonas maltophilia* infection among young children in a cardiac intensive care unit: a single institution experience. Pediatr. Cardiol. 36, 509–515. 10.1007/s00246-014-1041-0 25293429

[B5] Barbolla R. Catalano M. Orman B. E. Famiglietti A. Vay C. Smayevsky J.. (2004). Class 1 integrons increase trimethoprim-sulfamethoxazole MICs against epidemiologically unrelated *Stenotrophomonas maltophilia* isolates. Antimicrob. Agents Chemother. 48, 666–669. 10.1128/AAC.48.2.666-669.2004 14742234 PMC321565

[B6] Bassetti M. Della Siega P. Pecori D. Scarparo C. Righi E. (2015). Delafloxacin for the treatment of respiratory and skin infections. Expert Opin. Investig. Drugs 24, 433–442. 10.1517/13543784.2015.1005205 25604710

[B7] Berenbaum M. C. Yu V. L. Felegie T. P. (1983). Synergy with double and triple antibiotic combinations compared. J. Antimicrob. Chemother. 12, 555–563. 10.1093/jac/12.6.555 6662835

[B8] Betts J. W. Phee L. M. Woodford N. Wareham D. W. (2014). Activity of colistin in combination with tigecycline or rifampicin against multidrug-resistant *Stenotrophomonas maltophilia*. Eur. J. Clin. Microbiol. Infect. Dis. 33, 1565–1572. 10.1007/s10096-014-2101-3 24781003

[B9] Bouchillon S. K. Badal R. E. Hoban D. J. Hawser S. P. (2013). Antimicrobial susceptibility of inpatient urinary tract isolates of gram-negative bacilli in the United States: results from the study for monitoring antimicrobial resistance trends (SMART) program: 2009-2011. Clin. Ther. 35, 872–877. 10.1016/j.clinthera.2013.03.022 23623624

[B10] Brooke J. S. (2012). *Stenotrophomonas maltophilia*: an emerging global opportunistic pathogen. Clin. Microbiol. Rev. 25, 2–41. 10.1128/CMR.00019-11 22232370 PMC3255966

[B11] Brooke J. S. (2014). New strategies against *Stenotrophomonas maltophilia*: a serious worldwide intrinsically drug-resistant opportunistic pathogen. Expert. Rev. Anti. Infect. Ther. 12, 1–4. 10.1586/14787210.2014.864553 24308713

[B12] Cantón R. Valdezate S. Vindel A. Sánchez Del Saz B. Maíz L. Baquero F. (2003). Antimicrobial susceptibility profile of molecular typed cystic fibrosis *Stenotrophomonas maltophilia* isolates and differences with noncystic fibrosis isolates. Pediatr. Pulmonol. 35, 99–107. 10.1002/ppul.10216 12526070

[B13] Castanheira M. Mendes R. E. Jones R. N. (2014). Update on *Acinetobacter* species: mechanisms of antimicrobial resistance and contemporary *in vitro* activity of minocycline and other treatment options. Clin. Infect. Dis. 59(Suppl. 6), S367–S373. 10.1093/cid/ciu706 25371512

[B14] Chang Y. T. Lin C. Y. Lu P. L. Lai C. C. Chen T. C. Chen C. Y.. (2014). *Stenotrophomonas maltophilia* bloodstream infection: comparison between community-onset and hospital-acquired infections. J. Microbiol. Immunol. Infect. 47, 28–35. 10.1016/j.jmii.2012.08.014 23040236

[B15] Chen C. H. Huang C. C. Chung T. C. Hu R. M. Huang Y. W. Yang T. C. (2011). Contribution of resistance-nodulation-division efflux pump operon *sme*U1-V-W-U2-X to multidrug resistance of *Stenotrophomonas maltophilia*. Antimicrob. Agents Chemother. 55, 5826–5833. 10.1128/AAC.00317-11 21930878 PMC3232770

[B16] Chen Y. H. Lu P. L. Huang C. H. Liao C. H. Lu C. T. Chuang Y. C.. (2012). Trends in the susceptibility of clinically important resistant bacteria to tigecycline: results from the tigecycline *in vitro* surveillance in Taiwan study, 2006 to 2010. Antimicrob. Agents Chemother. 56, 1452–1457. 10.1128/AAC.06053-11 22203598 PMC3294947

[B17] Chen X. X. Lo Y. C. Su L. H. Chang C. L. (2014). Investigation of the case number of catheter-related bloodstream infection overestivated by the central line-associated bloodstream infection surveillance definition. J. Microbiol. Immunol. Infect. 10.1016/j.jmii.2014.03.006 [Epub ahead of print]. 24856425

[B18] Cho S. Y. Kang C. I. Kim J. Ha Y. E. Chung D. R. Lee N. Y.. (2014). Can levofloxacin be a useful alternative to trimethoprim-sulfamethoxazole for treating *Stenotrophomonas maltophilia* bacteremia? Antimicrob. Agents Chemother. 58, 581–583. 10.1128/AAC.01682-13 24126583 PMC3910801

[B19] Chow A. W. Wong J. Bartlett K. H. (1988). Synergistic interactions of ciprofloxacin and extended-spectrum beta-lactams or aminoglycosides against multiply drug-resistant *Pseudomonas maltophilia*. Antimicrob. Agents Chemother. 32, 782–784. 10.1128/AAC.32.5.782 3395107 PMC172276

[B20] Chung H. S. Hong S. G. Kim Y. R. Shin K. S. Whang D. H. Ahn J. Y.. (2013). Antimicrobial susceptibility of *Stenotrophomonas maltophilia* isolates from Korea, and the activity of antimicrobial combinations against the isolates. J. Korean Med. Sci. 28, 62–66. 10.3346/jkms.2013.28.1.62 23341713 PMC3546106

[B21] Chung H. S. Kim K. Hong S. S. Hong S. G. Lee K. Chong Y. (2015). The *sul1* gene in *Stenotrophomonas maltophilia* with high-level resistance to trimethoprim/sulfamethoxazole. Ann. Lab. Med. 35, 246–249. 10.3343/alm.2015.35.2.246 25729729 PMC4330177

[B22] Church D. Lloyd T. Peirano G. Pitout J. (2013). Antimicrobial susceptibility and combination testing of invasive *Stenotrophomonas maltophilia* isolates. Scand. J. Infect. Dis. 45, 265–270. 10.3109/00365548.2012.732240 23113657

[B23] Clinical Laboratory Standards Institute (CLSI) (2015). Performance Standards for Antimicrobial Susceptibility Testing; 25th Informational Supplement. CLSI Document M100-S25. Wayne, PA: CLSI.

[B24] Correia C. R. Ferreira S. T. Nunes P. (2014). *Stenotrophomonas maltophilia*: rare cause of meningitis. Pediatr. Int. 56, e21–e22. 10.1111/ped.12352 25252064

[B25] Crossman L. C. Gould V. C. Dow J. M. Vernikos G. S. Okazaki A. Sebaihia M.. (2008). The complete genome, comparative and functional analysis of *Stenotrophomonas maltophilia* reveals an organism heavily shielded by drug resistance determinants. Genome Biol. 9:R74. 10.1186/gb-2008-9-4-r74 18419807 PMC2643945

[B26] de Oliveira-Garcia D. Dall'agnol M. Rosales M. Azzuz A. C. Alcantara N. Martinez M. B.. (2003). Fimbriae and adherence of *Stenotrophomonas maltophilia* to epithelial cells and to abiotic surfaces. Cell Microbiol. 5, 625–636. 10.1046/j.1462-5822.2003.00306.x 12925132

[B27] del Toro M. D. Rodríguez-Bano J. Herrero M. Rivero A. García-Ordoñez M. A. Corzo J.. (2002). Clinical epidemiology of *Stenotrophomonas maltophilia* colonization and infection: a multicenter study. Medicine (Baltimore) 81, 228–239. 10.1097/00005792-200205000-00006 11997719

[B28] Diekema D. J. Pfaller M. A. Jones R. N. Doern G. V. Winokur P. L. Gales A. C.. (1999). Survey of bloodstream infections due to gram-negative bacilli: frequency of occurrence and antimicrobial susceptibility of isolates collected in the United States, Canada, and Latin America for the SENTRY Antimicrobial Surveillance Program, 1997. Clin. Infect. Dis. 29, 595–607. 10.1086/598640 10530454

[B29] Doern G. V. Jones R. N. Pfaller M. A. Kugler K. C. Beach M. L. (1999). Bacterial pathogens isolated from patients with skin and soft tissue infections: frequency of occurrence and antimicrobial susceptibility patterns from the SENTRY Antimicrobial Surveillance Program (United States and Canada, 1997). SENTRY Study Group (North America). Diagn. Microbiol. Infect. Dis. 34, 65–72. 10.1016/S0732-8893(98)00162-X 10342110

[B30] Entenza J. M. Moreillon P. (2009). Tigecycline in combination with other antimicrobials: a review of *in vitro*, animal and case report studies. Int. J. Antimicrob. Agents 34, 8.e1–8.e9. 10.1016/j.ijantimicag.2008.11.006 19162449

[B31] Falagas M. E. Kasiakou S. K. (2005). Colistin: the revival of polymyxins for the management of multidrug-resistant gram-negative bacterial infections. Clin. Infect. Dis. 40, 1333–1341. 10.1086/429323 15825037

[B32] Falagas M. E. Kastoris A. C. Vouloumanou E. K. Dimopoulos G. (2009a). Community-acquired *Stenotrophomonas maltophilia* infections: a systematic review. Eur. J. Clin. Microbiol. Infect. Dis. 28, 719–730. 10.1007/s10096-009-0709-5 19224257

[B33] Falagas M. E. Kastoris A. C. Vouloumanou E. K. Rafailidis P. I. Kapaskelis A. M. Dimopoulos G. (2009b). Attributable mortality of *Stenotrophomonas maltophilia* infections: a systematic review of the literature. Future Microbiol. 4, 1103–1109. 10.2217/fmb.09.84 19895214

[B34] Falagas M. E. Valkimadi P. E. Huang Y. T. Matthaiou D. K. Hsueh P. R. (2008). Therapeutic options for S*tenotrophomonas maltophilia* infections beyond co-trimoxazole: a systematic review. J Antimicrob. Chemother. 62, 889–894. 10.1093/jac/dkn301 18662945

[B35] Falagas M. E. Vardakas K. Z. Tsiveriotis K. P. Triarides N. A. Tansarli G. S. (2014). Effectiveness and safety of high-dose tigecycline-containing regimens for the treatment of severe bacterial infections. Int. J. Antimicrob. Agents 44, 1–7. 10.1016/j.ijantimicag.2014.01.006 24602499

[B36] Farrell D. J. Sader H. S. Flamm R. K. Jones R. N. (2014). Ceftolozane/tazobactam activity tested against Gram-negative bacterial isolates from hospitalised patients with pneumonia in US and European medical centres (2012). Int. J. Antimicrob. Agents 43, 533–539. 10.1016/j.ijantimicag.2014.01.032 24856078

[B37] Farrell D. J. Sader H. S. Jones R. N. (2010a). Antimicrobial susceptibilities of a worldwide collection of *Stenotrophomonas maltophilia* isolates tested against tigecycline and agents commonly used for *S. maltophilia* infections. Antimicrob. Agents Chemother. 54, 2735–2737. 10.1128/AAC.01774-09 20368399 PMC2876359

[B38] Farrell D. J. Turnidge J. D. Bell J. Sader H. S. Jones R. N. (2010b). The *in vitro* evaluation of tigecycline tested against pathogens isolated in eight countries in the Asia-Western Pacific region (2008). J. Infect. 60, 440–451. 10.1016/j.jinf.2010.03.024 20361999

[B39] Fedler K. A. Biedenbach D. J. Jones R. N. (2006a). Assessment of pathogen frequency and resistance patterns among pediatric patient isolates: report from the 2004 SENTRY Antimicrobial Surveillance Program on 3 continents. Diagn. Microbiol. Infect. Dis. 56, 427–436. 10.1016/j.diagmicrobio.2006.07.003 16938419

[B40] Fedler K. A. Jones R. N. Sader H. S. Fritsche T. R. (2006b). Activity of gatifloxacin tested against isolates from pediatric patients: report from the SENTRY Antimicrobial Surveillance Program (North America, 1998-2003). Diagn. Microbiol. Infect. Dis. 55, 157–164. 10.1016/j.diagmicrobio.2006.01.005 16529904

[B41] Felegie T. P. Yu V. L. Rumans L. W. Yee R. B. (1979). Susceptibility of *Pseudomonas maltophilia* to antimicrobial agents, singly and in combination. Antimicrob. Agents Chemother. 16, 833–837. 10.1128/AAC.16.6.833 533264 PMC352962

[B42] Fihman V. Le Monnier A. Corvec S. Jaureguy F. Tankovic J. Jacquier H.. (2012). *Stenotrophomonas maltophilia*–the most worrisome threat among unusual non-fermentative gram-negative bacilli from hospitalized patients: a prospective multicenter study. J. Infect. 64, 391–398. 10.1016/j.jinf.2012.01.001 22245400

[B43] Flores-Treviño S. Gutiérrez-Ferman J. L. Morfín-Otero R. Rodríguez-Noríega E. Estrada-Rívadeneyra D. Rivas-Morales C.. (2014). *Stenotrophomonas maltophilia* in Mexico: antimicrobial resistance, biofilm formation and clonal diversity. J. Med. Microbiol. 63, 1524–1530. 10.1099/jmm.0.074385-0 25165124

[B44] Fluit A. C. Schmitz F. J. Verhoef J. European S. P. G. (2001a). Frequency of isolation of pathogens from bloodstream, nosocomial pneumonia, skin and soft tissue, and urinary tract infections occurring in European patients. Eur. J. Clin. Microbiol. Infect. Dis. 20, 188–191. 10.1007/s100960100455 11347669

[B45] Fluit A. C. Verhoef J. Schmitz F. J. European S. P. (2001b). Frequency of isolation and antimicrobial resistance of gram-negative and gram-positive bacteria from patients in intensive care units of 25 European university hospitals participating in the European arm of the SENTRY Antimicrobial Surveillance Program 1997-1998. Eur. J. Clin. Microbiol. Infect. Dis. 20, 617–625. 10.1007/s10096-001-8078-8 11714042

[B46] Fujita J. Yamadori I. Xu G. Hojo S. Negayama K. Miyawaki H.. (1996). Clinical features of *Stenotrophomonas maltophilia* pneumonia in immunocompromised patients. Respir. Med. 90, 35–38. 10.1016/S0954-6111(96)90242-5 8857324

[B47] Gales A. C. Jones R. N. Forward K. R. Liñares J. Sader H. S. Verhoef J. (2001a). Emerging importance of multidrug-resistant *Acinetobacter* species and *Stenotrophomonas maltophilia* as pathogens in seriously ill patients: geographic patterns, epidemiological features, and trends in the SENTRY Antimicrobial Surveillance Program (1997-1999). Clin. Infect. Dis. 32(Suppl. 2), S104–S113. 10.1086/320183 11320451

[B48] Gales A. C. Jones R. N. Sader H. S. (2006). Global assessment of the antimicrobial activity of polymyxin B against 54 731 clinical isolates of Gram-negative bacilli: report from the SENTRY antimicrobial surveillance programme (2001-2004). Clin. Microbiol. Infect. 12, 315–321. 10.1111/j.1469-0691.2005.01351.x 16524407

[B49] Gales A. C. Reis A. O. Jones R. N. (2001b). Contemporary assessment of antimicrobial susceptibility testing methods for polymyxin B and colistin: review of available interpretative criteria and quality control guidelines. J. Clin. Microbiol. 39, 183–190. 10.1128/JCM.39.1.183-190.2001 11136768 PMC87699

[B50] Gales A. C. Sader H. H. Jones R. N. (2002). Respiratory tract pathogens isolated from patients hospitalized with suspected pneumonia in Latin America: frequency of occurrence and antimicrobial susceptibility profile: results from the SENTRY Antimicrobial Surveillance Program (1997-2000). Diagn. Microbiol. Infect. Dis. 44, 301–311. 10.1016/S0732-8893(02)00499-6 12493178

[B51] Gales A. C. Sader H. S. Fritsche T. R. (2008). Tigecycline activity tested against 11808 bacterial pathogens recently collected from US medical centers. Diagn. Microbiol. Infect. Dis. 60, 421–427. 10.1016/j.diagmicrobio.2007.10.017 18068934

[B52] García-León G. Hernández A. Hernando-Amado S. Alavi P. Berg G. Martínez J. L. (2014a). A function of SmeDEF, the major quinolone resistance determinant of *Stenotrophomonas maltophilia*, is the colonization of plant roots. Appl. Environ. Microbiol. 80, 4559–4565. 10.1128/AEM.01058-14 24837376 PMC4148791

[B53] García-León G. Ruiz de Alegría Puig C. García de la Fuente C. Martínez-Martínez L. Martínez J. L. Sánchez M. B. (2015). High-level quinolone resistance is associated with the overexpression of *smeVWX* in *Stenotrophomonas maltophilia* clinical isolates. Clin. Microbiol. Infect. 21, 464–467. 10.1016/j.cmi.2015.01.007 25753190

[B54] García-León G. Salgado F. Oliveros J. C. Sánchez M. B. Martínez J. L. (2014b). Interplay between intrinsic and acquired resistance to quinolones in *Stenotrophomonas maltophilia*. Environ. Microbiol. 16, 1282–1296. 10.1111/1462-2920.12408 24447641

[B55] Giamarellos-Bourboulis E. J. Karnesis L. Giamarellou H. (2002). Synergy of colistin with rifampin and trimethoprim/sulfamethoxazole on multidrug-resistant *Stenotrophomonas maltophilia*. Diagn. Microbiol. Infect. Dis. 44, 259–263. 10.1016/S0732-8893(02)00443-1 12493173

[B56] Gould V. C. Okazaki A. Avison M. B. (2013). Coordinate hyperproduction of SmeZ and SmeJK efflux pumps extends drug resistance in *Stenotrophomonas maltophilia*. Antimicrob. Agents Chemother. 57, 655–657. 10.1128/AAC.01020-12 23147729 PMC3535947

[B57] Guembe M. Cercenado E. Alcalá L. Marin M. Insa R. Bouza E. (2008). Evolution of antimicrobial susceptibility patterns of aerobic and facultative gram-negative bacilli causing intra-abdominal infections: results from the SMART studies 2003-2007. Rev. Esp. Quimioter. 21, 166–173. Retrieved from http://seq.es/seq/0214-3429/21/3/guembe.pdf 18792817

[B58] Gülmez D. Cakar A. Sener B. Karakaya J. Hasçelik G. (2010). Comparison of different antimicrobial susceptibility testing methods for *Stenotrophomonas maltophilia* and results of synergy testing. J. Infect. Chemother. 16, 322–328. 10.1007/s10156-010-0068-2 20449623

[B59] Gülmez D. Hasçelik G. (2005). *Stenotrophomonas maltophilia*: antimicrobial resistance and molecular typing of an emerging pathogen in a Turkish University Hospital. Clin. Microbiol. Infect. 11, 880–886. 10.1111/j.1469-0691.2005.01257.x 16216102

[B60] Harthan A. A. Heger M. L. (2013). *Stenotrophomonas* infection in a patient with glucose-6-phosphate dehydrogenase deficiency. J. Pediatr. Pharmacol. Ther. 18, 137–141. 10.5863/1551-6776-18.2.137 23798908 PMC3668942

[B61] Hernández A. Maté M. J. Sánchez-Díaz P. C. Romero A. Rojo F. Martínez J. L. (2009). Structural and functional analysis of SmeT, the repressor of the *Stenotrophomonas maltophilia* multidrug efflux pump SmeDEF. J. Biol. Chem. 284, 14428–14438. 10.1074/jbc.M809221200 19324881 PMC2682891

[B62] Hoban D. J. Biedenbach D. J. Mutnick A. H. Jones R. N. (2003). Pathogen of occurrence and susceptibility patterns associated with pneumonia in hospitalized patients in North America: results of the SENTRY Antimicrobial Surveillance Study (2000). Diagn. Microbiol. Infect. Dis. 45, 279–285. 10.1016/S0732-8893(02)00540-0 12730000

[B63] Hombach M. Bloemberg G. V. Böttger E. C. (2012). Effects of clinical breakpoint changes in CLSI guidelines 2010/2011 and EUCAST guidelines 2011 on antibiotic susceptibility test reporting of Gram-negative bacilli. J. Antimicrob. Chemother. 67, 622–632. 10.1093/jac/dkr524 22167240

[B64] Hornsey M. Longshaw C. Phee L. Wareham D. W. (2012). *In vitro* activity of telavancin in combination with colistin versus Gram-negative bacterial pathogens. Antimicrob. Agents Chemother. 56, 3080–3085. 10.1128/AAC.05870-11 22491686 PMC3370780

[B65] Hotta G. Matsumura Y. Kato K. Nakano S. Yunoki T. Yamamoto M.. (2014). Risk factors and outcomes of *Stenotrophomonas maltophilia* bacteraemia: a comparison with bacteraemia caused by *Pseudomonas aeruginosa* and *Acinetobacter* species. PLoS ONE 9:e112208. 10.1371/journal.pone.0112208 25375244 PMC4223050

[B66] Hu L. F. Chang X. Ye Y. Wang Z. X. Shao Y. B. Shi W.. (2011). *Stenotrophomonas maltophilia* resistance to trimethoprim/sulfamethoxazole mediated by acquisition of sul and dfrA genes in a plasmid-mediated class 1 integron. Int. J. Antimicrob. Agents 37, 230–234. 10.1016/j.ijantimicag.2010.10.025 21296557

[B67] Hu L. F. Gao L. P. Ye Y. Chen X. Zhou X. T. Yang H. F.. (2014). Susceptibility of *Stenotrophomonas maltophilia* clinical strains in China to antimicrobial combinations. J. Chemother. 26, 282–286. 10.1179/1973947814Y.0000000168 24588423

[B68] Hu R. M. Liao S. T. Huang C. C. Huang Y. W. Yang T. C. (2012). An inducible fusaric acid tripartite efflux pump contributes to the fusaric acid resistance in *Stenotrophomonas maltophilia*. PLoS ONE 7:e51053. 10.1371/journal.pone.0051053 23236431 PMC3517613

[B69] Huang C. H. Lai C. C. Chen Y. H. Hsueh P. R. (2015). The potential role of nemonoxacin for treatment of common infections. Expert. Opin. Pharmacother. 16, 263–270. 10.1517/14656566.2015.978288 25529577

[B70] Huang Y. W. Hu R. M. Chu F. Y. Lin H. R. Yang T. C. (2013a). Characterization of a major facilitator superfamily (MFS) tripartite efflux pump EmrCABsm from *Stenotrophomonas maltophilia*. J. Antimicrob. Chemother. 68, 2498–2505. 10.1093/jac/dkt250 23794602

[B71] Huang Y. W. Hu R. M. Yang T. C. (2013b). Role of the pcm-tolCsm operon in the multidrug resistance of *Stenotrophomonas maltophilia*. J. Antimicrob. Chemother. 68, 1987–1993. 10.1093/jac/dkt148 23629016

[B72] Huang Y. W. Lin C. W. Hu R. M. Lin Y. T. Chung T. C. Yang T. C. (2010). AmpN-AmpG operon is essential for expression of L1 and L2 beta-lactamases in *Stenotrophomonas maltophilia*. Antimicrob. Agents Chemother. 54, 2583–2589. 10.1128/AAC.01283-09 20385866 PMC2876420

[B73] Huang Y. W. Liou R. S. Lin Y. T. Huang H. H. Yang T. C. (2014). A linkage between SmeIJK efflux pump, cell envelope integrity, and sigmaE-mediated envelope stress response in *Stenotrophomonas maltophilia*. PLoS ONE 9:e111784. 10.1371/journal.pone.0111784 25390933 PMC4229105

[B74] Irifune K. Ishida T. Shimoguchi K. Ohtake J. Tanaka T. Morikawa N.. (1994). Pneumonia caused by *Stenotrophomonas maltophilia* with a mucoid phenotype. J. Clin. Microbiol. 32, 2856–2857. 7852587 10.1128/jcm.32.11.2856-2857.1994 PMC264175

[B75] Jang T. N. Wang F. D. Wang L. S. Liu C. Y. Liu I. M. (1992). Xanthomonas maltophilia bacteremia: an analysis of 32 cases. J. Formos. Med. Assoc. 91, 1170–1176. 1363639

[B76] Jean S. S. Lee W. S. Bai K. J. Lam C. Hsu C. W. Yu K. W.. (2015). Relationship between the distribution of cefepime minimum inhibitory concentrations and detection of extended-spectrum β-lactamase production among clinically important *Enterobacteriaceae* isolates obtained from patients in intensive care units in Taiwan: results from the Surveillance of Multicenter Antimicrobial Resistance in Taiwan (SMART) in 2007. J. Microbiol. Immunol. Infect. 48, 85–91. 10.1016/j.jmii.2013.07.002 23973410

[B77] Jones R. N. (2010). Microbial etiologies of hospital-acquired bacterial pneumonia and ventilator-associated bacterial pneumonia. Clin. Infect. Dis. 51(Suppl. 1), S81–S87. 10.1086/653053 20597676

[B78] Jones R. N. Croco M. A. Kugler K. C. Pfaller M. A. Beach M. L. (2000). Respiratory tract pathogens isolated from patients hospitalized with suspected pneumonia: frequency of occurrence and antimicrobial susceptibility patterns from the SENTRY Antimicrobial Surveillance Program (United States and Canada, 1997). Diagn. Microbiol. Infect. Dis. 37, 115–125. 10.1016/S0732-8893(00)00115-2 10863106

[B79] Jones R. N. Croco M. A. Pfaller M. A. Beach M. L. Kugler K. C. SENTRY Antimicrobial Surveillance Program Participants. (1999a). Antimicrobial activity evaluations of gatifloxacin, a new fluoroquinolone: contemporary pathogen results from a global antimicrobial resistance surveillance program (SENTRY, 1997). Clin. Microbiol. Infect. 5, 540–546. 10.1111/j.1469-0691.1999.tb00432.x 11851706

[B80] Jones R. N. Kugler K. C. Pfaller M. A. Winokur P. L. (1999b). Characteristics of pathogens causing urinary tract infections in hospitals in North America: results from the SENTRY Antimicrobial Surveillance Program, 1997. Diagn. Microbiol. Infect. Dis. 35, 55–63. 10.1016/S0732-8893(98)00158-8 10529882

[B81] Jones R. N. Pfaller M. A. Marshall S. A. Hollis R. J. Wilke W. W. (1997). Antimicrobial activity of 12 broad-spectrum agents tested against 270 nosocomial blood stream infection isolates caused by non-enteric gram-negative bacilli: occurrence of resistance, molecular epidemiology, and screening for metallo-enzymes. Diagn. Microbiol. Infect. Dis. 29, 187–192. 10.1016/S0732-8893(97)81808-1 9401811

[B82] Jones R. N. Sader H. S. Beach M. L. (2003). Contemporary *in vitro* spectrum of activity summary for antimicrobial agents tested against 18569 strains non-fermentative Gram-negative bacilli isolated in the SENTRY Antimicrobial Surveillance Program (1997-2001). Int. J. Antimicrob. Agents 22, 551–556. 10.1016/S0924-8579(03)00245-0 14659650

[B83] Kerr K. G. Hawkey P. M. Child J. A. Norfolk D. R. Anderson A. W. (1990). *Pseudomonas maltophilia* infections in neutropenic patients and the use of imipenem. Postgrad. Med. J. 66:1090. 10.1136/pgmj.66.782.1090 2084668 PMC2429775

[B84] Khan I. U. Mirza I. A. Ikram A. Ali S. Hussain A. Ghafoor T. (2014). *In vitro* activity of fosfomycin tromethamine against extended spectrum beta-lactamase producing urinary tract bacteria. J. Coll. Physicians. Surg. Pak. 24, 914–917. 25523727

[B85] Kim T. Chong Y. P. Park S. Y. Jeon M. H. Choo E. J. Chung J. W.. (2014). Risk factors for hospital-acquired pneumonia caused by carbapenem-resistant Gram-negative bacteria in critically ill patients: a multicenter study in Korea. Diagn. Microbiol. Infect. Dis. 78, 457–461. 10.1016/j.diagmicrobio.2013.08.011 24462178

[B86] King P. Lomovskaya O. Griffith D. C. Burns J. L. Dudley M. N. (2010). *In vitro* pharmacodynamics of levofloxacin and other aerosolized antibiotics under multiple conditions relevant to chronic pulmonary infection in cystic fibrosis. Antimicrob. Agents Chemother. 54, 143–148. 10.1128/AAC.00248-09 19805554 PMC2798504

[B87] Krueger T. S. Clark E. A. Nix D. E. (2001). *In vitro* susceptibility of *Stenotrophomonas maltophilia* to various antimicrobial combinations. Diagn. Microbiol. Infect. Dis. 41, 71–78. 10.1016/S0732-8893(01)00281-4 11687317

[B88] Labarca J. A. Leber A. L. Kern V. L. Territo M. C. Brankovic L. E. Bruckner D. A.. (2000). Outbreak of *Stenotrophomonas maltophilia* bacteremia in allogenic bone marrow transplant patients: role of severe neutropenia and mucositis. Clin. Infect. Dis. 30, 195–197. 10.1086/313591 10619754

[B89] Lai C. C. Lee K. Y. Lin S. W. Chen Y. H. Kuo H. Y. Hung C. C.. (2014a). Nemonoxacin (TG-873870) for treatment of community-acquired pneumonia. Expert Rev. Anti. Infect. Ther. 12, 401–417. 10.1586/14787210.2014.894881 24579813

[B90] Lai C. H. Chi C. Y. Chen H. P. Chen T. L. Lai C. J. Fung C. P.. (2004). Clinical characteristics and prognostic factors of patients with *Stenotrophomonas maltophilia* bacteremia. J. Microbiol. Immunol. Infect. 37, 350–358. 15599467

[B91] Lai C. H. Wong W. W. Chin C. Huang C. K. Lin H. H. Chen W. F.. (2006). Central venous catheter-related *Stenotrophomonas maltophilia* bacteraemia and associated relapsing bacteraemia in haematology and oncology patients. Clin. Microbiol. Infect. 12, 986–991. 10.1111/j.1469-0691.2006.01511.x 16961635

[B92] Lai W. A. Chen S. F. Tsai N. W. Chang W. N. Lu C. H. Chuang Y. C.. (2014b). Non-cephalosporin-susceptible, glucose non-fermentative Gram-negative bacilli meningitis in post-neurosurgical adults: clinical characteristics and therapeutic outcome. Clin. Neurol. Neurosurg. 116, 61–66. 10.1016/j.clineuro.2013.10.020 24287342

[B93] Lee M. R. Wang H. C. Yang C. Y. Lin C. K. Kuo H. Y. Ko J. C.. (2014). Clinical characteristics and outcomes of patients with pleural infections due to *Stenotrophomonas maltophilia* at a medical center in Taiwan, 2004-2012. Eur. J. Clin. Microbiol. Infect. Dis. 33, 1143–1148. 10.1007/s10096-014-2060-8 24458500

[B94] Lee Y. L. Chen Y. S. Toh H. S. Huang C. C. Liu Y. M. Ho C. M.. (2012). Antimicrobial susceptibility of pathogens isolated from patients with complicated intra-abdominal infections at five medical centers in Taiwan that continuously participated in the Study for Monitoring Antimicrobial Resistance Trends (SMART) from 2006 to 2010. Int. J. Antimicrob. Agents 40(Suppl.), S29–S36. 10.1016/s0924-8579(12)70007-9 22749056

[B95] Li X. Z. Zhang L. Mckay G. A. Poole K. (2003). Role of the acetyltransferase AAC(6')-Iz modifying enzyme in aminoglycoside resistance in *Stenotrophomonas maltophilia*. J. Antimicrob. Chemother. 51, 803–811. 10.1093/jac/dkg148 12654758

[B96] Li X. Z. Zhang L. Poole K. (2002). SmeC, an outer membrane multidrug efflux protein of *Stenotrophomonas maltophilia*. Antimicrob. Agents Chemother. 46, 333–343. 10.1128/AAC.46.2.333-343.2002 11796339 PMC127032

[B97] Lin C. W. Huang Y. W. Hu R. M. Yang T. C. (2014a). SmeOP-TolCSm efflux pump contributes to the multidrug resistance of *Stenotrophomonas maltophilia*. Antimicrob. Agents Chemother. 58, 2405–2408. 10.1128/AAC.01974-13 24395237 PMC4023765

[B98] Lin C. W. Lin H. C. Huang Y. W. Chung T. C. Yang T. C. (2011). Inactivation of mrcA gene derepresses the basal-level expression of L1 and L2 beta-lactamases in *Stenotrophomonas maltophilia*. J. Antimicrob. Chemother. 66, 2033–2037. 10.1093/jac/dkr276 21719470

[B99] Lin Y. T. Huang Y. W. Chen S. J. Chang C. W. Yang T. C. (2015). SmeYZ efflux pump of *Stenotrophomonas maltophilia* contributes to drug resistance, virulence-related characteristics, and virulence to mice. Antimicrob. Agents Chemother. 59, 4067–4073. 10.1128/AAC.00372-15 25918140 PMC4468721

[B100] Lin Y. T. Huang Y. W. Liou R. S. Chang Y. C. Yang T. C. (2014b). MacABCsm, an ABC-type tripartite efflux pump of *Stenotrophomonas maltophilia* involved in drug resistance, oxidative and envelope stress tolerances and biofilm formation. J. Antimicrob. Chemother. 69, 3221–3226. 10.1093/jac/dku317 25139838

[B101] Lindberg F. Westman L. Normark S. (1985). Regulatory components in *Citrobacter freundii* ampC beta-lactamase induction. Proc. Natl. Acad. Sci. U.S.A. 82, 4620–4624. 10.1073/pnas.82.14.4620 2991883 PMC390437

[B102] Liu Y. M. Chen Y. S. Toh H. S. Huang C. C. Lee Y. L. Ho C. M. (2012). *In vitro* susceptibilities of non-*Enterobacteriaceae* isolates from patients with intra-abdominal infections in the Asia-Pacific region from 2003 to 2010: results from the Study for Monitoring Antimicrobial Resistance Trends (SMART). Int. J. Antimicrob. Agents 40(Suppl.), S11–S17. 10.1016/s0924-8579(12)70004-3 22749053

[B103] Livermore D. M. Hope R. Brick G. Lillie M. Reynolds R. BSAC Working Parties on Resistance Surveillance. (2008). Non-susceptibility trends among *Pseudomonas aeruginosa* and other non-fermentative Gram-negative bacteria from bacteraemias in the UK and Ireland, 2001-06. J. Antimicrob. Chemother. 62(Suppl. 2), ii55–ii63. 10.1093/jac/dkn352 18819980

[B104] Livermore D. M. Mushtaq S. Warner M. Woodford N. (2014). Comparative *in vitro* activity of sulfametrole/trimethoprim and sulfamethoxazole/trimethoprim and other agents against multiresistant Gram-negative bacteria. J. Antimicrob. Chemother. 69, 1050–1056. 10.1093/jac/dkt455 24257317

[B105] Lodge J. M. Minchin S. D. Piddock L. J. Busby S. J. (1990). Cloning, sequencing and analysis of the structural gene and regulatory region of the *Pseudomonas aeruginosa* chromosomal ampC beta-lactamase. Biochem. J. 272, 627–631. 10.1042/bj2720627 2125210 PMC1149754

[B106] Looney W. J. Narita M. Mühlemann K. (2009). *Stenotrophomonas maltophilia*: an emerging opportunist human pathogen. Lancet Infect. Dis. 9, 312–323. 10.1016/S1473-3099(09)70083-0 19393961

[B107] Lu P. L. Liu Y. C. Toh H. S. Lee Y. L. Liu Y. M. Ho C. M.. (2012). Epidemiology and antimicrobial susceptibility profiles of Gram-negative bacteria causing urinary tract infections in the Asia-Pacific region: 2009-2010 results from the Study for Monitoring Antimicrobial Resistance Trends (SMART). Int. J. Antimicrob. Agents 40(Suppl.), S37–S43. 10.1016/s0924-8579(12)70008-0 22749057

[B108] Macleod D. L. Barker L. M. Sutherland J. L. Moss S. C. Gurgel J. L. Kenney T. F.. (2009). Antibacterial activities of a fosfomycin/tobramycin combination: a novel inhaled antibiotic for bronchiectasis. J. Antimicrob. Chemother. 64, 829–836. 10.1093/jac/dkp282 19679597 PMC2740636

[B109] Magret M. Lisboa T. Martin-Loeches I. Máñez R. Nauwynck M. Wrigge H.. (2011). Bacteremia is an independent risk factor for mortality in nosocomial pneumonia: a prospective and observational multicenter study. Crit. Care 15, R62. 10.1186/cc10036 21324159 PMC3221995

[B110] Mathai D. Lewis M. T. Kugler K. C. Pfaller M. A. Jones R. N. SENTRY Participants Group (North America). (2001). Antibacterial activity of 41 antimicrobials tested against over 2773 bacterial isolates from hospitalized patients with pneumonia: I–results from the SENTRY Antimicrobial Surveillance Program (North America, 1998). Diagn. Microbiol. Infect. Dis. 39, 105–116. 10.1016/S0732-8893(00)00234-0 11248523

[B111] Memish Z. A. Shibl A. M. Kambal A. M. Ohaly Y. A. Ishaq A. Livermore D. M. (2012). Antimicrobial resistance among non-fermenting Gram-negative bacteria in Saudi Arabia. J. Antimicrob. Chemother. 67, 1701–1705. 10.1093/jac/dks091 22461312

[B112] Meyer E. Schwab F. Gastmeier P. Rueden H. Daschner F. D. Jonas D. (2006). *Stenotrophomonas maltophilia* and antibiotic use in German intensive care units: data from Project SARI (Surveillance of Antimicrobial Use and Antimicrobial Resistance in German Intensive Care Units). J. Hosp. Infect. 64, 238–243. 10.1016/j.jhin.2006.07.006 16979794

[B113] Micozzi A. Venditti M. Monaco M. Friedrich A. Taglietti F. Santilli S.. (2000). Bacteremia due to *Stenotrophomonas maltophilia* in patients with hematologic malignancies. Clin. Infect. Dis. 31, 705–711. 10.1086/314043 11017819

[B114] Milne K. E. Gould I. M. (2012). Combination antimicrobial susceptibility testing of multidrug-resistant *Stenotrophomonas maltophilia* from cystic fibrosis patients. Antimicrob. Agents Chemother. 56, 4071–4077. 10.1128/AAC.00072-12 22585220 PMC3421579

[B115] Morrissey I. Hackel M. Badal R. Bouchillon S. Hawser S. Biedenbach D. (2013). A review of ten years of the Study for Monitoring Antimicrobial Resistance Trends (SMART) from 2002 to 2011. Pharmaceuticals (Basel) 6, 1335–1346. 10.3390/ph6111335 24287460 PMC3854014

[B116] Moskowitz S. M. Garber E. Chen Y. Clock S. A. Tabibi S. Miller A. K.. (2010). Colistin susceptibility testing: evaluation of reliability for cystic fibrosis isolates of *Pseudomonas aeruginosa* and *Stenotrophomonas maltophilia*. J. Antimicrob. Chemother. 65, 1416–1423. 10.1093/jac/dkq131 20430789 PMC2882871

[B117] Moya B. Dötsch A. Juan C. Blázquez J. Zamorano L. Haussler S.. (2009). Beta-lactam resistance response triggered by inactivation of a nonessential penicillin-binding protein. PLoS Pathog. 5:e1000353. 10.1371/journal.ppat.1000353 19325877 PMC2654508

[B118] Nicodemo A. C. Araujo M. R. Ruiz A. S. Gales A. C. (2004). *In vitro* susceptibility of *Stenotrophomonas maltophilia* isolates: comparison of disc diffusion, Etest and agar dilution methods. J. Antimicrob. Chemother. 53, 604–608. 10.1093/jac/dkh128 14973153

[B119] Nicodemo A. C. Paez J. I. (2007). Antimicrobial therapy for *Stenotrophomonas maltophilia infections*. Eur. J. Clin. Microbiol. Infect. Dis. 26, 229–237. 10.1007/s10096-007-0279-3 17334747

[B120] Ning B. T. Zhang C. M. Liu T. Ye S. Yang Z. H. Chen Z. J. (2013). Pathogenic analysis of sputum from ventilator-associated pneumonia in a pediatric intensive care unit. Exp. Ther. Med. 5, 367–371. 10.3892/etm.2012.757 23251300 PMC3524271

[B121] Nord C. E. Wadström T. Wretlind B. (1974). Synergistic effect of combinations of sulfamethoxazole, trimethoprim, and colistin against *Pseudomonas maltophilia* and *Pseudomonas cepacia*. Antimicrob. Agents Chemother. 6, 521–523. 10.1128/AAC.6.4.521 4143759 PMC444683

[B122] O'Riordan W. Mehra P. Manos P. Kingsley J. Lawrence L. Cammarata S. (2015). A randomized phase 2 study comparing two doses of delafloxacin with tigecycline in adults with complicated skin and skin-structure infections. Int. J. Infect. Dis. 30, 67–73. 10.1016/j.ijid.2014.10.009 25448332

[B123] Okazaki A. Avison M. B. (2007). Aph(3′)-IIc, an aminoglycoside resistance determinant from *Stenotrophomonas maltophilia*. Antimicrob. Agents Chemother. 51, 359–360. 10.1128/AAC.00795-06 17088477 PMC1797691

[B124] Okazaki A. Avison M. B. (2008). Induction of L1 and L2 beta-lactamase production in *Stenotrophomonas maltophilia* is dependent on an AmpR-type regulator. Antimicrob. Agents Chemother. 52, 1525–1528. 10.1128/AAC.01485-07 18212097 PMC2292540

[B125] Paez J. I. Costa S. F. (2008). Risk factors associated with mortality of infections caused by *Stenotrophomonas maltophilia*: a systematic review. J. Hosp. Infect. 70, 101–108. 10.1016/j.jhin.2008.05.020 18621440

[B126] Page M. G. Dantier C. Desarbre E. Gaucher B. Gebhardt K. Schmitt-Hoffmann A. (2011). *In vitro* and *in vivo* properties of BAL30376, a beta-lactam and dual beta-lactamase inhibitor combination with enhanced activity against Gram-negative Bacilli that express multiple beta-lactamases. Antimicrob. Agents Chemother. 55, 1510–1519. 10.1128/AAC.01370-10 21245441 PMC3067176

[B127] Papadakis K. A. Vartivarian S. E. Vassilaki M. E. Anaissie E. J. (1995). *Stenotrophomonas maltophilia*: an unusual cause of biliary sepsis. Clin. Infect. Dis. 21, 1032–1034. 10.1093/clinids/21.4.1032 8645796

[B128] Perez P. N. Ramirez M. A. Fernandez J. A. De Guevara L. L. (2014). A patient presenting with cholangitis due to *Stenotrophomonas maltophilia* and *Pseudomonas aeruginosa* successfully treated with intrabiliary colistin. Infect. Dis. Rep. 6:5147. 10.4081/idr.2014.5147 25002957 PMC4083296

[B129] Personne Y. Curtis M. A. Wareham D. W. Waite R. D. (2014). Activity of the type I signal peptidase inhibitor MD3 against multidrug-resistant Gram-negative bacteria alone and in combination with colistin. J. Antimicrob. Chemother. 69, 3236–3243. 10.1093/jac/dku309 25134721

[B130] Pien C. J. Kuo H. Y. Chang S. W. Chen P. R. Yeh H. W. Liu C. C.. (2015). Risk factors for levofloxacin resistance in *Stenotrophomonas maltophilia* from respiratory tract in a regional hospital. J. Microbiol. Immunol. Infect. 48, 291–295. 10.1016/j.jmii.2013.09.005 24239064

[B131] Pfaller M. A. Jones R. N. Doern G. V. Kugler K. (1998). Bacterial pathogens isolated from patients with bloodstream infection: frequencies of occurrence and antimicrobial susceptibility patterns from the SENTRY antimicrobial surveillance program (United States and Canada, 1997). Antimicrob. Agents Chemother. 42, 1762–1770. 9661018 10.1128/aac.42.7.1762 PMC105680

[B132] Pompilio A. Crocetta V. Confalone P. Nicoletti M. Petrucca A. Guarnieri S.. (2010). Adhesion to and biofilm formation on IB3-1 bronchial cells by *Stenotrophomonas maltophilia* isolates from cystic fibrosis patients. BMC Microbiol. 10:102. 10.1186/1471-2180-10-102 20374629 PMC2858031

[B133] Poulos C. D. Matsumura S. O. Willey B. M. Low D. E. McGeer A. (1995). *In vitro* activities of antimicrobial combinations against *Stenotrophomonas (Xanthomonas) maltophilia*. Antimicrob. Agents Chemother. 39, 2220–2223. 10.1128/AAC.39.10.2220 8619571 PMC162918

[B134] Putman M. Van Veen H. W. Konings W. N. (2000). Molecular properties of bacterial multidrug transporters. Microbiol. Mol. Biol. Rev. 64, 672–693. 10.1128/MMBR.64.4.672-693.2000 11104814 PMC99009

[B135] Ratjen A. Yau Y. Wettlaufer J. Matukas L. Zlosnik J. E. Speert D. P.. (2015). *In vitro* efficacy of high-dose tobramycin against *Burkholderia cepacia* complex and *Stenotrophomonas maltophilia* isolates from cystic fibrosis patients. Antimicrob. Agents Chemother. 59, 711–713. 10.1128/AAC.04123-14 25348526 PMC4291427

[B136] Rattanaumpawan P. Ussavasodhi P. Kiratisin P. Aswapokee N. (2013). Epidemiology of bacteremia caused by uncommon non-fermentative gram-negative bacteria. BMC Infect. Dis. 13:167. 10.1186/1471-2334-13-167 23566113 PMC3636083

[B137] Renteria M. I. Biedenbach D. J. Bouchillon S. K. Hoban D. J. Raghubir N. Sajben P.. (2014). *In vitro* activity of tigecycline against isolates collected from complicated skin and skin structure infections and intra-abdominal infections in Africa and Middle East countries: TEST 2007-2012. Diagn. Microbiol. Infect. Dis. 79, 54–59. 10.1016/j.diagmicrobio.2014.01.017 24582580

[B138] Rhee J. Y. Choi J. Y. Choi M. J. Song J. H. Peck K. R. Ko K. S. (2013). Distinct groups and antimicrobial resistance of clinical *Stenotrophomonas maltophilia* complex isolates from Korea. J. Med. Microbiol. 62, 748–753. 10.1099/jmm.0.053355-0 23429694

[B139] Rizek C. Ferraz J. R. Van der Heijden I. M. Giudice M. Mostachio A. K. Paez J.. (2015). *In vitro* activity of potential old and new drugs against multidrug-resistant gram-negatives. J. Infect. Chemother. 21, 114–117. 10.1016/j.jiac.2014.10.009 25456893

[B140] Rodríguez C. H. Nastro M. Calvo J. L. Fariña M. E. Dabos L. Famiglietti A. (2014). *In vitro* activity of colistin against *Stenotrophomonas maltophilia*. J. Glob. Antimicrob. Resist. 2, 316–317. 10.1016/j.jgar.2014.04.004 27873694

[B141] Sader H. S. Farrell D. J. Flamm R. K. Jones R. N. (2014a). Antimicrobial susceptibility of Gram-negative organisms isolated from patients hospitalised with pneumonia in US and European hospitals: results from the SENTRY Antimicrobial Surveillance Program, 2009-2012. Int. J. Antimicrob. Agents 43, 328–334. 10.1016/j.ijantimicag.2014.01.007 24630306

[B142] Sader H. S. Farrell D. J. Flamm R. K. Jones R. N. (2014b). Variation in potency and spectrum of tigecycline activity against bacterial strains from U.S. medical centers since its approval for clinical use (2006 to 2012). Antimicrob. Agents Chemother. 58, 2274–2280. 10.1128/AAC.02684-13 24492361 PMC4023762

[B143] Sader H. S. Flamm R. K. Jones R. N. (2013). Tigecycline activity tested against antimicrobial resistant surveillance subsets of clinical bacteria collected worldwide (2011). Diagn. Microbiol. Infect. Dis. 76, 217–221. 10.1016/j.diagmicrobio.2013.02.009 23522845

[B144] Sader H. S. Jones R. N. (2005). Antimicrobial susceptibility of uncommonly isolated non-enteric Gram-negative bacilli. Int. J. Antimicrob. Agents 25, 95–109. 10.1016/j.ijantimicag.2004.10.002 15664479

[B145] Sader H. S. Jones R. N. Dowzicky M. J. Fritsche T. R. (2005a). Antimicrobial activity of tigecycline tested against nosocomial bacterial pathogens from patients hospitalized in the intensive care unit. Diagn. Microbiol. Infect. Dis. 52, 203–208. 10.1016/j.diagmicrobio.2005.05.002 16105565

[B146] Sader H. S. Jones R. N. Gales A. C. Silva J. B. Pignatari A. C. SENTRY Participants Group (Latin America). (2004). SENTRY antimicrobial surveillance program report: Latin American and Brazilian results for 1997 through 2001. Braz. J. Infect. Dis. 8, 25–79. 10.1590/S1413-86702004000100004 15286878

[B147] Sader H. S. Jones R. N. Gales A. C. Winokur P. Kugler K. C. Pfaller M. A.. (1998). Antimicrobial susceptibility patterns for pathogens isolated from patients in Latin American medical centers with a diagnosis of pneumonia: analysis of results from the SENTRY Antimicrobial Surveillance Program (1997). SENTRY Latin America Study Group. Diagn. Microbiol. Infect. Dis. 32, 289–301. 10.1016/S0732-8893(98)00124-2 9934546

[B148] Sader H. S. Jones R. N. Stilwell M. G. Dowzicky M. J. Fritsche T. R. (2005b). Tigecycline activity tested against 26,474 bloodstream infection isolates: a collection from 6 continents. Diagn. Microbiol. Infect. Dis. 52, 181–186. 10.1016/j.diagmicrobio.2005.05.005 16105562

[B149] Safdar A. Rolston K. V. (2007). *Stenotrophomonas maltophilia*: changing spectrum of a serious bacterial pathogen in patients with cancer. Clin. Infect. Dis. 45, 1602–1609. 10.1086/522998 18190323

[B150] Saiman L. Chen Y. Gabriel P. S. Knirsch C. (2002). Synergistic activities of macrolide antibiotics against *Pseudomonas aeruginosa, Burkholderia cepacia, Stenotrophomonas maltophilia*, and *Alcaligenes xylosoxidans* isolated from patients with cystic fibrosis. Antimicrob. Agents Chemother. 46, 1105–1107. 10.1128/AAC.46.4.1105-1107.2002 11897598 PMC127106

[B151] Samonis G. Karageorgopoulos D. E. Maraki S. Levis P. Dimopoulou D. Spernovasilis N. A.. (2012). *Stenotrophomonas maltophilia* infections in a general hospital: patient characteristics, antimicrobial susceptibility, and treatment outcome. PLoS ONE 7:e37375. 10.1371/journal.pone.0037375 22624022 PMC3356252

[B152] San Gabriel P. Zhou J. Tabibi S. Chen Y. Trauzzi M. Saiman L. (2004). Antimicrobial susceptibility and synergy studies of *Stenotrophomonas maltophilia* isolates from patients with cystic fibrosis. Antimicrob. Agents Chemother. 48, 168–171. 10.1128/AAC.48.1.168-171.2004 14693535 PMC310154

[B153] Sanchez M. B. Hernandez A. Martinez J. L. (2009). *Stenotrophomonas maltophilia* drug resistance. Future Microbiol. 4, 655–660. 10.2217/fmb.09.45 19659422

[B154] Sánchez M. B. Hernández A. Rodríguez-Martínez J. M. Martínez-Martínez L. Martínez J. L. (2008). Predictive analysis of transmissible quinolone resistance indicates *Stenotrophomonas maltophilia* as a potential source of a novel family of Qnr determinants. BMC Microbiol. 8:148. 10.1186/1471-2180-8-148 18793450 PMC2556341

[B155] Sanchez M. B. Martinez J. L. (2010). SmQnr contributes to intrinsic resistance to quinolones in *Stenotrophomonas maltophilia*. Antimicrob. Agents Chemother. 54, 580–581. 10.1128/AAC.00496-09 19841154 PMC2798493

[B156] Sánchez M. B. Martínez L. (2015). The efflux pump SmeDEF contributes to trimethoprim/sulfamethoxazole resistance in *Stenotrophomonas maltophilia*. Antimicrob. Agents Chemother. 59, 4347–4348. 10.1128/AAC.00714-15 25918144 PMC4468665

[B157] Shimizu K. Kikuchi K. Sasaki T. Takahashi N. Ohtsuka M. Ono Y.. (2008). Smqnr, a new chromosome-carried quinolone resistance gene in *Stenotrophomonas maltophilia*. Antimicrob. Agents Chemother. 52, 3823–3825. 10.1128/AAC.00026-08 18644963 PMC2565915

[B158] Sood S. Vaid V. K. Bhartiya H. (2013). Meningitis due to *Stenotrophomonas maltophilia* after a Neurosurgical Procedure. J. Clin. Diagn. Res. 7, 1696–1697. 10.7860/jcdr/2013/5614.3248 24086879 PMC3782936

[B159] Stein G. E. Babinchak T. (2013). Tigecycline: an update. Diagn. Microbiol. Infect. Dis. 75, 331–336. 10.1016/j.diagmicrobio.2012.12.004 23357291

[B160] Streit J. M. Jones R. N. Sader H. S. Fritsche T. R. (2004). Assessment of pathogen occurrences and resistance profiles among infected patients in the intensive care unit: report from the SENTRY Antimicrobial Surveillance Program (North America, 2001). Int. J. Antimicrob. Agents 24, 111–118. 10.1016/j.ijantimicag.2003.12.019 15288308

[B161] Tada T. Miyoshi-Akiyama T. Dahal R. K. Mishra S. K. Shimada K. Ohara H.. (2014). Identification of a novel 6′-N-aminoglycoside acetyltransferase, AAC(6′)-Iak, from a multidrug-resistant clinical isolate of *Stenotrophomonas maltophilia*. Antimicrob. Agents Chemother. 58, 6324–6327. 10.1128/AAC.03354-14 25092711 PMC4187913

[B162] Talfan A. Mounsey O. Charman M. Townsend E. Avison M. B. (2013). Involvement of mutation in ampD I, mrcA, and at least one additional gene in beta-lactamase hyperproduction in *Stenotrophomonas maltophilia*. Antimicrob. Agents Chemother. 57, 5486–5491. 10.1128/AAC.01446-13 23979761 PMC3811264

[B163] Tan R. Liu J. Li M. Huang J. Sun J. Qu H. (2014). Epidemiology and antimicrobial resistance among commonly encountered bacteria associated with infections and colonization in intensive care units in a university affiliated hospital in Shanghai. J. Microbiol. Immunol. Infect. 47, 87–94. 10.1016/j.jmii.2012.11.006 23357606

[B164] Tekçe Y. T. Erbay A. Cabadak H. Sen S. (2012). Tigecycline as a therapeutic option in *Stenotrophomonas maltophilia* infections. J. Chemother. 24, 150–154. 10.1179/1120009X12Z.00000000022 22759759

[B165] Toleman M. A. Bennett P. M. Bennett D. M. Jones R. N. Walsh T. R. (2007). Global emergence of trimethoprim/sulfamethoxazole resistance in *Stenotrophomonas maltophilia* mediated by acquisition of *sul* genes. Emerg. Infect. Dis. 13, 559–565. 10.3201/eid1304.061378 17553270 PMC2725981

[B166] Valdezate S. Vindel A. Loza E. Baquero F. Cantón R. (2001). Antimicrobial susceptibilities of unique *Stenotrophomonas maltophilia* clinical strains. Antimicrob. Agents Chemother. 45, 1581–1584. 10.1128/AAC.45.5.1581-1584.2001 11302834 PMC90512

[B167] Valdezate S. Vindel A. Saéz-Nieto J. A. Baquero F. Cantón R. (2005). Preservation of topoisomerase genetic sequences during *in vivo* and *in vitro* development of high-level resistance to ciprofloxacin in isogenic *Stenotrophomonas maltophilia* strains. J. Antimicrob. Chemother. 56, 220–223. 10.1093/jac/dki182 15928010

[B168] Valenza G. Tappe D. Turnwald D. Frosch M. König C. Hebestreit H.. (2008). Prevalence and antimicrobial susceptibility of microorganisms isolated from sputa of patients with cystic fibrosis. J. Cyst. Fibros. 7, 123–127. 10.1016/j.jcf.2007.06.006 17693140

[B169] Vartivarian S. Anaissie E. Bodey G. Sprigg H. Rolston K. (1994). A changing pattern of susceptibility of *Xanthomonas maltophilia* to antimicrobial agents: implications for therapy. Antimicrob. Agents Chemother. 38, 624–627. 10.1128/AAC.38.3.624 8203865 PMC284510

[B170] Vartivarian S. E. Anaissie E. J. Kiwan E. N. Papadakis K. A. (2000). The clinical spectrum of *Stenotrophomonas (Xanthomonas) maltophilia* respiratory infection. Semin. Respir. Crit. Care Med. 21, 349–355. 10.1055/s-2000-9859 16088746

[B171] Vartivarian S. E. Papadakis K. A. Anaissie E. J. (1996). Stenotrophomonas (Xanthomonas) maltophilia urinary tract infection. A disease that is usually severe and complicated. Arch. Intern. Med. 156, 433–435. 10.1001/archinte.1996.00440040111012 8607729

[B172] Victor M. A. Arpi M. Bruun B. Jønsson V. Hansen M. M. (1994). *Xanthomonas maltophilia* bacteremia in immunocompromised hematological patients. Scand. J. Infect. Dis. 26, 163–170. 10.3109/00365549409011780 8036472

[B173] Visalli M. A. Jacobs M. R. Appelbaum P. C. (1998). Activities of three quinolones, alone and in combination with extended-spectrum cephalosporins or gentamicin, against *Stenotrophomonas maltophilia*. Antimicrob. Agents Chemother. 42, 2002–2005. 9687397 10.1128/aac.42.8.2002 PMC105723

[B174] Walkty A. Adam H. Baxter M. Denisuik A. Lagacé-Wiens P. Karlowsky J. A.. (2014). *In vitro* activity of plazomicin against 5,015 gram-negative and gram-positive clinical isolates obtained from patients in canadian hospitals as part of the CANWARD study, 2011-2012. Antimicrob. Agents Chemother. 58, 2554–2563. 10.1128/AAC.02744-13 24550325 PMC3993217

[B175] Wang C. H. Lin J. C. Lin H. A. Chang F. Y. Wang N. C. Chiu S. K.. (2014a). Comparisons between patients with trimethoprim-sulfamethoxazole-susceptible and trimethoprim-sulfamethoxazole-resistant *Stenotrophomonas maltophilia* monomicrobial bacteremia: A 10-year retrospectivestudy. J. Microbiol. Immunol. Infect. 10.1016/j.jmii.2014.06.005 [Epub ahead of print] 25081988

[B176] Wang Y. L. Scipione M. R. Dubrovskaya Y. Papadopoulos J. (2014b). Monotherapy with fluoroquinolone or trimethoprim-sulfamethoxazole for treatment of *Stenotrophomonas maltophilia* infections. Antimicrob. Agents Chemother. 58, 176–182. 10.1128/AAC.01324-13 24145530 PMC3910778

[B177] Waters V. (2012). New treatments for emerging cystic fibrosis pathogens other than *Pseudomonas*. Curr. Pharm. Des. 18, 696–725. 10.2174/138161212799315939 22229574

[B178] Wood G. C. Underwood E. L. Croce M. A. Swanson J. M. Fabian T. C. (2010). Treatment of recurrent *Stenotrophomonas maltophilia* ventilator-associated pneumonia with doxycycline and aerosolized colistin. Ann. Pharmacother. 44, 1665–1668. 10.1345/aph.1P217 20736426

[B179] Wu H. Wang J. T. Shiau Y. R. Wang H. Y. Lauderdale T. L. Chang S. C.. (2012). A multicenter surveillance of antimicrobial resistance on *Stenotrophomonas maltophilia* in Taiwan. J. Microbiol. Immunol. Infect. 45, 120–126. 10.1016/j.jmii.2011.09.028 22154599

[B180] Wu K. Yau Y. C. Matukas L. Waters V. (2013). Biofilm compared to conventional antimicrobial susceptibility of *Stenotrophomonas maltophilia* Isolates from cystic fibrosis patients. Antimicrob. Agents Chemother. 57, 1546–1548. 10.1128/AAC.02215-12 23295930 PMC3591893

[B181] Wu Y. Shao Z. (2014). High-dosage tigecycline for *Stenotrophomonas maltophilia* bacteremia. Chin. Med. J. (Engl.) 127:3199. 10.3760/cma.j.issn.0366-6999.20140364 25189974

[B182] Yang Q. Wang H. Chen M. Ni Y. Yu Y. Hu B.. (2010). Surveillance of antimicrobial susceptibility of aerobic and facultative Gram-negative bacilli isolated from patients with intra-abdominal infections in China: the 2002-2009 Study for Monitoring Antimicrobial Resistance Trends (SMART). Int. J. Antimicrob. Agents 36, 507–512. 10.1016/j.ijantimicag.2010.09.001 21036547

[B183] Yang T. C. Huang Y. W. Hu R. M. Huang S. C. Lin Y. T. (2009). AmpDI is involved in expression of the chromosomal L1 and L2 beta-lactamases of *Stenotrophomonas maltophilia*. Antimicrob. Agents Chemother. 53, 2902–2907. 10.1128/AAC.01513-08 19414581 PMC2704650

[B184] Yu V. L. Felegie T. P. Yee R. B. Pasculle A. W. Taylor F. H. (1980). Synergistic interaction *in vitro* with use of three antibiotics simultaneously against *Pseudomonas maltophilia*. J. Infect. Dis. 142, 602–607. 10.1093/infdis/142.4.602 7441019

[B185] Zhanel G. G. Adam H. J. Baxter M. R. Fuller J. Nichol K. A. Denisuik A. J.. (2013). Antimicrobial susceptibility of 22746 pathogens from Canadian hospitals: results of the CANWARD 2007-11 study. J. Antimicrob. Chemother. 68(Suppl. 1), i7–i22. 10.1093/jac/dkt022 23587781

[B186] Zhanel G. G. Adam H. J. Low D. E. Blondeau J. Decorby M. Karlowsky J. A.. (2011). Antimicrobial susceptibility of 15,644 pathogens from Canadian hospitals: results of the CANWARD 2007-2009 study. Diagn. Microbiol. Infect. Dis. 69, 291–306. 10.1016/j.diagmicrobio.2010.10.025 21353957

[B187] Zhanel G. G. Decorby M. Adam H. Mulvey M. R. Mccracken M. Lagacé-Wiens P.. (2010). Prevalence of antimicrobial-resistant pathogens in Canadian hospitals: results of the Canadian Ward Surveillance Study (CANWARD 2008). Antimicrob. Agents Chemother. 54, 4684–4693. 10.1128/AAC.00469-10 20805395 PMC2976152

[B188] Zhanel G. G. Decorby M. Nichol K. A. Wierzbowski A. Baudry P. J. Karlowsky J. A.. (2008). Antimicrobial susceptibility of 3931 organisms isolated from intensive care units in Canada: Canadian National Intensive Care Unit Study, 2005/2006. Diagn. Microbiol. Infect. Dis. 62, 67–80. 10.1016/j.diagmicrobio.2008.04.012 18513913

